# Cyclins and Cyclin‐Dependent Kinases: Structure, Biological Functions, and Innovative Targeting Strategies in Cancer

**DOI:** 10.1002/mco2.70898

**Published:** 2026-08-16

**Authors:** Suya Zheng, Zhipeng Shen, Yuanfang Wu, Xingze Huang, Zhongyuan Zhang, Jiangyu Liu, Qichun Wei, Wenhao Chen, Ye Chen, Liang Xu

**Affiliations:** ^1^ Department of Radiation Oncology (Key Laboratory of Cancer Prevention and Intervention China National Ministry of Education) The Second Affiliated Hospital Zhejiang University School of Medicine Hangzhou China; ^2^ Department of Neurosurgery Children's Hospital Zhejiang University School of Medicine, National Clinical Research Center For Children and Adolescents’ Health and Diseases Hangzhou China; ^3^ Pediatric Cancer Research Center National Clinical Research Center for Children and Adolescents’ Health and Diseases Hangzhou China; ^4^ Institute of Biochemistry College of Life Sciences Zhejiang University Hangzhou China; ^5^ Zhejiang Provincial Clinical Research Center For Cancer Hangzhou China; ^6^ Department of Orthopedic Surgery Children's Hospital Zhejiang University School of Medicine National Clinical Research Center for Children and Adolescents’ Health and Diseases Hangzhou China; ^7^ Cancer Center Zhejiang University Hangzhou China

**Keywords:** CDK, protein degrader, targeted protein degradation, TCIP, transcriptional kinase

## Abstract

Cyclins and cyclin‐dependent kinases (CDKs) are frequently dysregulated in human cancers and represent compelling therapeutic targets. Beyond their well‐recognized roles in cell cycle control, CDK/cyclin complexes orchestrate diverse oncogenic processes, including transcription, genome maintenance, epigenetics, metabolism, and immune regulation. Deciphering the multifaceted biology of CDK/cyclin will provide valuable insights and rationales for the development of CDK/cyclin‐targeting strategies and modalities. While the clinical success of CDK4/6 inhibitors has validated CDKs as druggable targets, further efforts are urgently needed to target other CDKs and cyclins. This review critically evaluates recent mechanistic advances in CDK/cyclin biology and their pathological dysregulation across malignancies. We analyze the paradigm shift from conventional enzymatic inhibition toward proximity‐induced modulation. Specifically, we highlight emerging approaches including proteolysis‐targeting chimeras, HSP90‐mediated targeting chimeras, hydrophobic tagging, molecular glues, and autophagy‐tethering compounds that achieve selective elimination of CDKs or their cyclin partners. In parallel, we summarize strategies designed to redistribute CDK complexes and rewire transcription without enzymatic ablation, referred to as chemical inducers of proximity and transcriptional/epigenetic modulators. By integrating fundamental CDK/cyclin biology with pharmacological innovation in targeted protein degradation and kinase reprogramming, this review provides a timely roadmap for the CDK/cyclin research field and expands the frontiers of CDK/cyclin‐targeted cancer therapy.

## Graphical abstract

This work synthesizes recent insights into the pathological roles of cyclins and cyclin‐dependent kinases (CDKs) across human cancers, highlights state‐of‐the‐art innovative approaches (especially targeted degradation and redistribution of CDK/cyclin proteins) for cancer therapy, and outlines future directions for CDK/cyclin‐related biomedical research. Deepening our molecular understanding of CDK/cyclin biology, coupled with continued therapeutic innovation, will ultimately accelerate the development of next‐generation precision medicines.

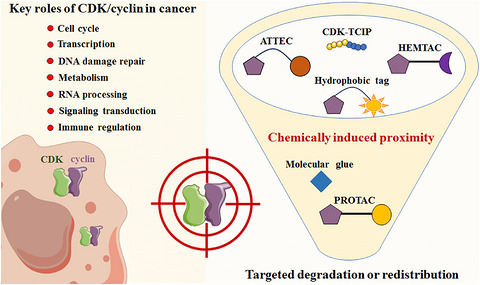

## 1 Introduction

Cyclin‐dependent kinases (CDKs) are serine/threonine protein kinases that are widely recognized as crucial regulators of the cell cycle from yeast to humans. The initial discovery and early characterization of CDK1 (Cdc2 in yeast) and its cyclin partner (in sea urchin) were undertaken by Leland H. Hartwell, R. Timothy Hunt, and Paul M. Nurse, who jointly received the 2001 Nobel Prize in Physiology or Medicine [1]. Since the first isolation of human CDK1, 21 CDK genes and an even larger number of cyclin genes have been identified in the human genome; the latter typically act as CDK dimerizing partners and regulatory subunits. CDKs are typically grouped into three primary classes [2, 3]: cell cycle‐regulatory CDKs (such as CDK1, CDK2, CDK3, CDK4, and CDK6), transcription‐related CDKs (CDK7, CDK8, CDK9, CDK10, CDK11, CDK12, CDK13, and CDK19), and atypical CDKs (including CDK5, CDK14, CDK15, CDK16, CDK17, CDK18, and CDK20). This classification, albeit a simplification, effectively captures the essential contributions of CDKs and their partnering cyclins. Recent studies have established that CDKs contribute significantly beyond cell cycle progression, with essential functions in DNA replication, DNA repair, transcription, RNA splicing, RNA polyadenylation, signal transduction, translation, and immune modulation [4, 5, 6, 7, 8]. Moreover, kinase‐independent roles of CDKs have been recognized [9].

CDKs and their partner cyclins are frequently dysregulated in human cancer. For instance, CDK4 and CDK6 are bona fide drivers of various cancers. Overexpression or hyperactivation of CDK4 or CDK6 is frequently observed in liposarcoma, esophageal adenocarcinoma, glioblastoma, and breast invasive carcinoma. Genomic amplifications of CDK2, CDK4, CDK6, CCND1/2/3, and CCNE1/2 were recurrently detected in cancer samples, affecting more than 17% of cancer patients (Table S1). These CDKs and cyclins enforce cell cycle regulation, particularly transition from the G1 phase to the S phase. Furthermore, high‐throughput genetic screening assays have revealed that most CDKs and cyclins are essential for cell viability in diverse types of cancers. Consequently, targeting these proteins for cancer therapy has been a key focus of pharmaceutical research [10].

To date, a variety of CDK inhibitors have been reported, acting through mechanisms like competitive inhibition, allosteric inhibition, or covalent binding. The initial breakthrough in CDK‐targeted therapy was the approval of palbociclib, a dual CDK4/6 inhibitor, for the treatment of hormone receptor‐positive, human epidermal growth factor receptor 2 (HER2)‐negative advanced or metastatic breast cancer. Since then, a growing list of CDK4/6 inhibitors (e.g., ribociclib, abemaciclib, dalpiciclib, tibremciclib, lerociclib, fovinaciclib, and trilaciclib), as well as the more recent CDK2/4/6 inhibitor culmerciclib and the CDK2/4/6/9 inhibitor bireociclib have also gained approval for targeted cancer therapy [11, 12, 13, 14, 15, 16]. However, the translational progress of other CDK inhibitors like dinaciclib and SNS‐032 faced significant challenges due to dose‐limiting toxicities and insufficient target specificity. Complementing traditional small‐molecule approaches, targeted protein degradation and subcellular relocalization strategies are gaining traction as promising strategies for the development of novel anticancer therapeutics [3, 17].

To advance CDK/cyclin‐targeted biomedical research, this review builds upon recent progress in the biology of CDK/cyclin in cancer and synthesizes emerging insights into their pathological functions. By tracking and summarizing the latest developments in molecular design and drug discovery, this article also outlines current frontiers in the CDK/cyclin‐targeting agents, including proteolysis‐targeting chimera (PROTAC), heat shock protein 90 (HSP90)‐mediated targeting chimera (HEMTAC), hydrophobic tagging (HyT), molecular glue, autophagy‐tethering compound (ATTEC), and relocation‐targeting chimera. It further highlights targeted degradation and redistribution of CDKs as two emerging paradigms for the development of next‐generation cancer therapeutics, and discusses key challenges, trends, and future directions in this field.

## 2 Structural Organization and Regulatory Mechanisms of CDK/Cyclin Complexes

The functional classification of CDKs into three primary classes reflects an increasing demand for regulatory precision and complexity in human cells. Similarly, cyclins are also clustered into three main categories: canonical (cyclin A/B/D/E), transcription‐associated (cyclin C/H/K/L/Q/T), and atypical (e.g., cyclin G/I/J/Y and CABLES1/2) [18]. Since the resolution of first CDK structure (CDK2), an accumulating body of structural studies has shed light on the structural organization and regulatory mechanisms of CDK/cyclin complexes [19, 20, 21].

### 2.1 Core Structural Organization of CDKs and Cyclins

**FIGURE 1:**
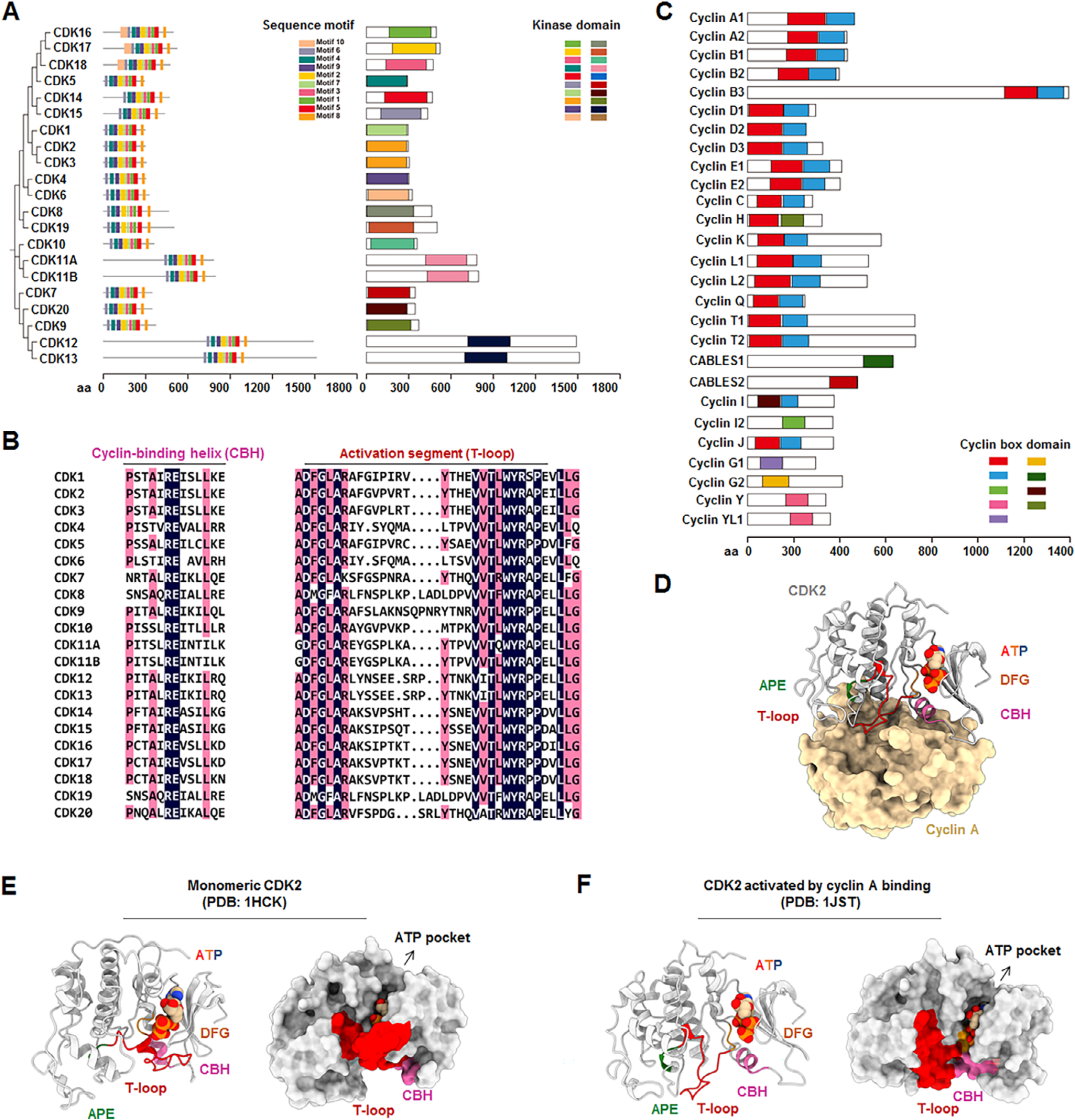
Structural organization of CDKs and cyclins. (A) Conserved kinase domain of human CDKs. Amino acid sequences of the CDKs are analyzed using the MEME Suite (classic mode; protein alphabet; site distribution: zoops; maximum motifs: 10). Kinase domain annotations are from the Conserved Domain Database (CDD). (B) Cyclin binding helix (CBH) and T‐loop sequence alignment across human CDK family members. (C) Conserved cyclin box domain of human cyclins. Cyclin box fold annotations are from the CDD. (D) CDK2/cyclin A interaction interface. Representative three‐dimensional structure of the CDK2/cyclin A complex (PDB: 1JST). APE, Ala‐Pro‐Glu motif. (E and F) Conformational activation of CDK2 upon cyclin A binding. Structural rearrangements accompanying cyclin binding are illustrated. Arrow indicates the ATP pocket.

Regardless of specialized roles, human CDKs share a conserved protein kinase catalytic domain (approximately 300 amino acids), which remains relatively invariant even when flanked by diverse N‐ or C‐terminal extensions (Figure 1A). This “catalytic core” adopts the characteristic eukaryotic protein kinase fold, organized into an N‐terminal lobe rich in β‐sheet (N‐lobe), a larger C‐terminal lobe rich in α‐helix (C‐lobe), and a deep cleft between the two lobes that forms the ATP‐binding and catalytic pocket [22]. A highly conserved α‐helix (cyclin‐binding helix [CBH]) within the N‐lope of the CDK kinase domain plays a critical role in cyclin binding (Figure 1B). Based on the motifs in CBH, CDKs are further classified into subgroups, including PSTAIRE (CDK1/2/3), PFTAIRE (CDK14/15), PCTAIRE (CDK16/17/18), PITSLRE (CDK11A/B), and others. The C‐lobe of CDK features a long activation segment (also known as T‐loop or activation loop), beginning with the conserved Asp–Phe–Gly (DFG) motif (also called the magnesium positioning loop) and ending at the αF‐helix (Figure 1B). The middle portion of the T‐loop possesses the greatest sequence diversity and flexibility within the kinase core and typically contains a phosphorylation site involved in allosteric regulation. Other highly conserved elements downstream of T‐loop include the P+1 loop, which serves as a docking site for the substrate residue backbone and the side chain of the residue following the phosphorylation site, as well as the Ala–Pro–Glu motif, which ensures structural integrity and kinase activity via intramolecular interactions mediated by a salt bridge [22].

In contrast to CDKs, cyclins generally share very little sequence homology but are structurally defined by the presence of one or two cyclin box domains (CBDs) (Figure 1C). Each CBD is organized as a compact, five‐α‐helical globular domain. The CBD is responsible for CDK interaction; more specifically, the N‐terminal CBD assumes this role when a second C‐terminal CBD is arranged in tandem, sparing the C‐terminal CBDs for regulatory or substrate‐docking functions. In some cyclin‐like proteins lacking canonical CBDs, such as CDK5R1, alternative domains with similar folds (e.g., CBD‐like domain) are employed to activate CDK partners. The CBD also presents a hydrophobic patch on its surface, which cyclins use to recognize and recruit CDK substrates containing specific docking motifs, such as the RxL motif [23, 24]. Such short linear motif‐mediated cyclin‐substrate docking represents a general theme that enables dynamic, context‐dependent substrate recognition and regulation. With regard to the sequences outside their CBDs, canonical cyclins involved in cell cycle control differ significantly from those involved in transcription (e.g., cyclin C/K/T), particularly in the length of the downstream portion and the orientation of secondary structures. These differences may underlie their mechanistic distinctions in functional complex assembly and substrate engagement [25].

### 2.2 Canonical Activation Mechanisms of CDK/Cyclin Complexes

The interaction between the CBH of a CDK and the CBD of a cyclin typically involves a large surface interface and subsequently induces a series of conformational changes in the CDK that are essential for its catalytic activity (Figure 1D–F). Indeed, monomeric CDK appears inactive, as its T‐loop blocks the entrance to the catalytic cleft in the free kinase. CDK is activated through cyclin association, phosphorylation, magnesium binding, and interactions with additional regulators [26, 27].

In the canonical model of CDK activation, exemplified by the seminal studies of CDK2/cyclin complexes [28, 29], the allosteric binding of a cyclin partner alleviates the blockade of the catalytic cleft, thereby partially activating the kinase function. In the meantime, this binding reorientates the DFG motif for magnesium positioning, reorganizes the CDK catalytic cleft to form the ATP‐binding site, and aligns residues required for catalysis, rendering the complex catalytically competent. Conformational responses of CDKs to cyclin binding are also coordinated with the phosphorylation of a conserved threonine residue located in its T‐loop, catalyzed by the CDK‐activating kinase (CAK; a trimeric complex of CDK7, cyclin H, and MAT1). Cushing et al. revealed that CAK recognizes its substrates through reciprocal binding of the C‐lobe of its CDK7 subunit to the C‐lobe of the CDK substrate, strengthened by additional interactions between their respective N‐lobes, generating a pseudo‐symmetrical kinase‐kinase interface analogous to a head‐to‐head dimer. The properties of different CDK/cyclin complexes, including the flexibility of the CDK T‐loop, influence substrate preference and phosphorylation kinetics of CAK [30]. The phosphorylated threonine residue in T‐loop then acts as a structural hub, coordinating positively charged residues to rearrange the active site and tether the T‐loop conformation for peptide substrate adoption. Consequently, the orientation of peptide substrates is dictated by the precise conformation of the phosphorylated T‐loop, with T/S–P–X–K/R as the canonical consensus CDK substrate motif [31]. Notably, CDK1 retains a locally more flexible T‐loop; such structural plasticity may accommodate diverse substrate sequences beyond the canonical motif and compensate for the functions of other CDKs [20, 32, 33]. Differences in the order of ATP loading and cyclin binding during CDK activation may influence its activation kinetics, as suggested by the slow activation of CDK4/cyclin D and the fast CDK2/cyclin E [34].

### 2.3 Noncanonical Activation Mechanisms of CDK/Cyclin Complexes

While the two‐step activation model of CDKs (i.e., canonical cyclin binding followed by T‐loop phosphorylation) is experimentally supported by studies of CDK2‐, CDK3‐, and CDK9‐related complexes [28, 29, 35], a growing number of reports have also revealed alternative strategies for CDK activation (comprehensive reviews are available elsewhere [27, 36]). These noncanonical activation mechanisms include accessory protein binding (e.g., CDK8/cyclin C/MED12, CDK16/cyclin Y/14‐3‐3, CDK7/cyclin H/MAT1, CDK4/cyclin D1/p27^Kip1^, CDK12/cyclin K/CDC73, CDK5/CDK5R1 p25 fragment) [37, 38, 39, 40, 41], cyclin N‐terminus engagement (e.g., CDK4/cyclin D) [42], and ordered T‐loop phosphorylation (e.g., CAK activity toward non‐CDK substrates) [43]. Thus, despite the general requirement of CDK/cyclin interaction for full catalytic activity, the structural basis of CDK activation varies significantly among different CDK/cyclin pairs. Further elucidation of the regulatory mechanisms underlying CDK/cyclin activation will guide the development of novel and selective therapeutic modalities targeting these complexes [44].

## 3 Dysregulation of CDKs and Their Partner Cyclins in Cancer Genome

**FIGURE 2:**
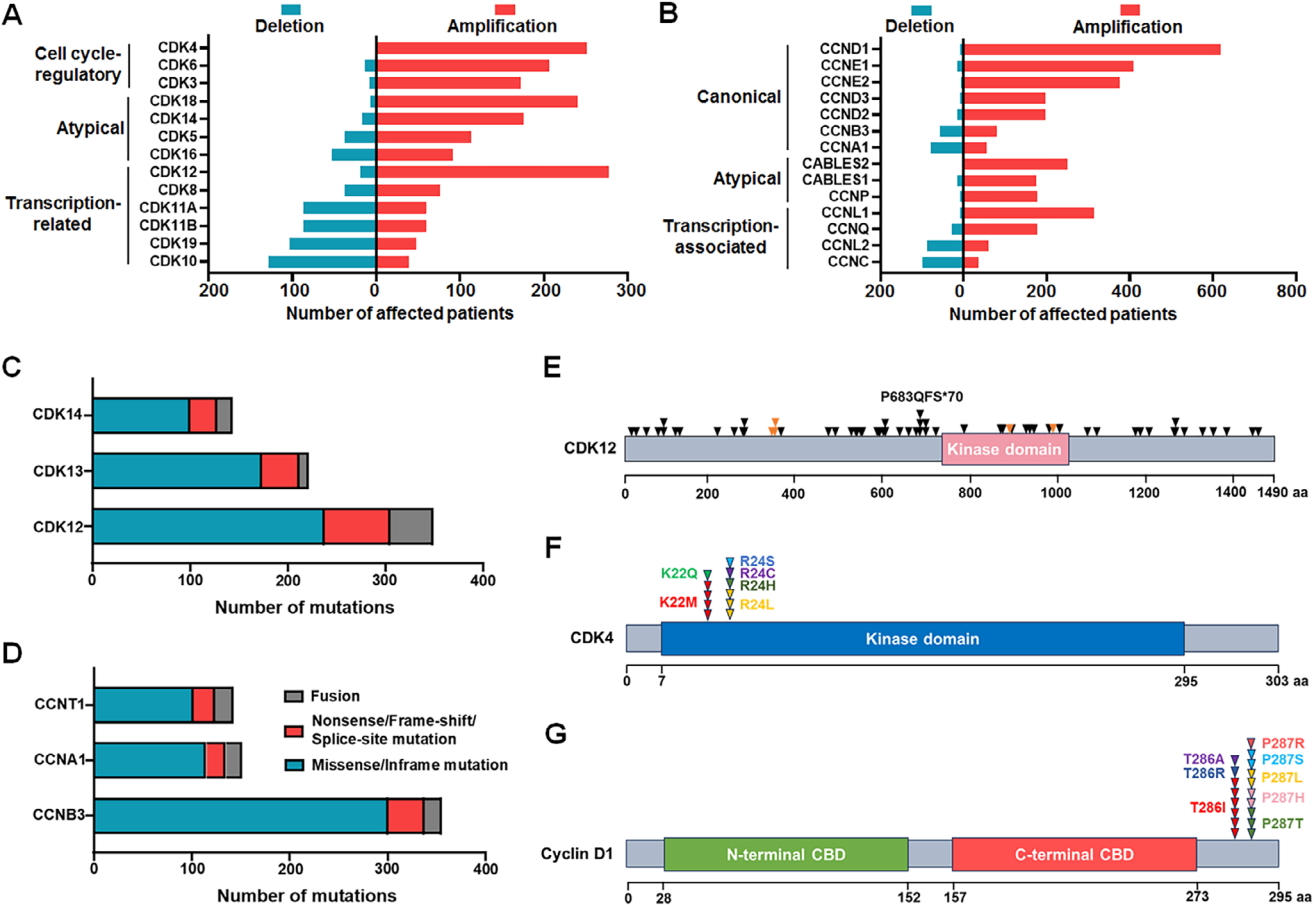
Genomic insults of CDKs and cyclins in human cancers. (A) CDK genes with recurrent copy number variations in 10967 cancer specimens within The Cancer Genome Atlas (TCGA) cohort. Genes with incidence rate over 1% were plotted. (B) Cyclin genes with recurrent copy number variations among the cancer specimens of TCGA cohort. Genes with incidence rate over 1% were plotted. (C) CDK genes with recurrent somatic mutations (frequency > 1%) among the cancer specimens of TCGA cohort. (D) Cyclin genes with recurrent somatic mutations (frequency > 1%). (E) Damaging mutations affecting CDK12. (F and G) Hot spot mutations affecting CDK4 and cyclin D1. In (E–G), each triangle represents a single mutational event. Nonsense mutations and frameshift indels are marked in black; splice site mutations are indicated in orange; nonsynonymous mutations are shown in other colors.

Based on pan‐cancer analysis via cBioPortal platform [45], genomic alterations of CDK and cyclin family genes were interrogated (Figure 2). Copy number variations and somatic mutations were evident for most members. Of note, CDK3, CDK4, CDK6, CDK12, CDK14, CDK18, CCND1/2/3, CCNE1/2, CCNL1, and CABLES1/2 were among the most recurrently amplified genes within their families in cancer samples, while CDK10, CDK19, and CCNC were deleted in a number of specimens (Figure 2A,B). CCND1, CDK4, and CDK6 amplifications represent well‐recognized oncogenic driver events across both solid cancers (e.g., sarcoma, esophageal cancer, head and neck squamous cell carcinoma, and breast cancer) and hematopoietic malignancies (e.g., lymphoma and leukemia). CDK4 and MDM2 coamplification represents a hallmark genomic feature of well‐differentiated and dedifferentiated liposarcoma. Selective dysregulation of a specific member of CDK4/6 and D‐type cyclins during cancer development may underscore context‐dependent oncogenic susceptibility involving lineage‐specific transcriptional programs and environmental factors.

CDK12, CDK13, CDK14, CCNT1, CCNA1, and CCNB3 were among the most frequently mutated CDKs and cyclins (Figure 2C,D). Remarkably, truncating mutations, splice site mutations, and structural variations constitute over 30% of CDK12 mutations (Figure 2E). Notably, CDK12 is recurrently inactivated in about 3–4% of serous ovarian adenocarcinomas and about 7% of metastatic castration‑resistant prostate cancers, consistently associated with increased aggressiveness and a genomic instability phenotype characterized by widespread tandem duplications [46, 47, 48]. CDK13 is mutated in about 4% of cutaneous melanomas, with an enrichment of dominant‐negative mutations in the kinase domain [49]. Several hotspot mutations were noted including CDK4 (R24C/L/H/S, K22M/Q) and CCND1 (P287S/T/L/R/H, T286I/A/R) (Figure 2F,G and Table S2), although their precise functions await further characterization.

Besides, several cancer‐promoting chromosomal translocations have been reported. For instance, BCOR::CCNB3 fusions are frequent in a subtype of undifferentiated round cell sarcomas [50]. Pathogenic translocations leading to IGH::CCND1, IGH::CCND2 or IGH::CCND3 juxtaposition are reported in the setting of B cell malignancies [51]. Furthermore, it is important to mention that nearly half of cancer patients are affected by genetic lesions resulting in loss of function of genes encoding CDK inhibitory proteins such like TP53, RB1, CDKN2A, and CDKN2B. Together, CDK/cyclin pathways are heavily dysregulated in cancer.

## 4 Functions of CDKs and Cyclins in Cancer

CDK/cyclin proteins have emerged as versatile signaling hubs in cancer. This section dissects their current functional landscape, organized around three main pillars (cell cycle‐regulatory, transcription‐associated, and atypical CDKs and their cyclin partners) and spanning a wide spectrum of oncogenic processes, including cell cycle control, transcriptional regulation, RNA processing, genome maintenance, signal transduction, epigenetic remodeling, metabolic reprogramming, and immune modulation. An improved understanding of both their canonical functions and their expanding functional complexity will guide the development of optimal therapeutic strategies and pharmacological interventions.

### 4.1 Functions of Cell Cycle‐Regulatory CDKs and the Associated Cyclins in Cancer

**FIGURE 3:**
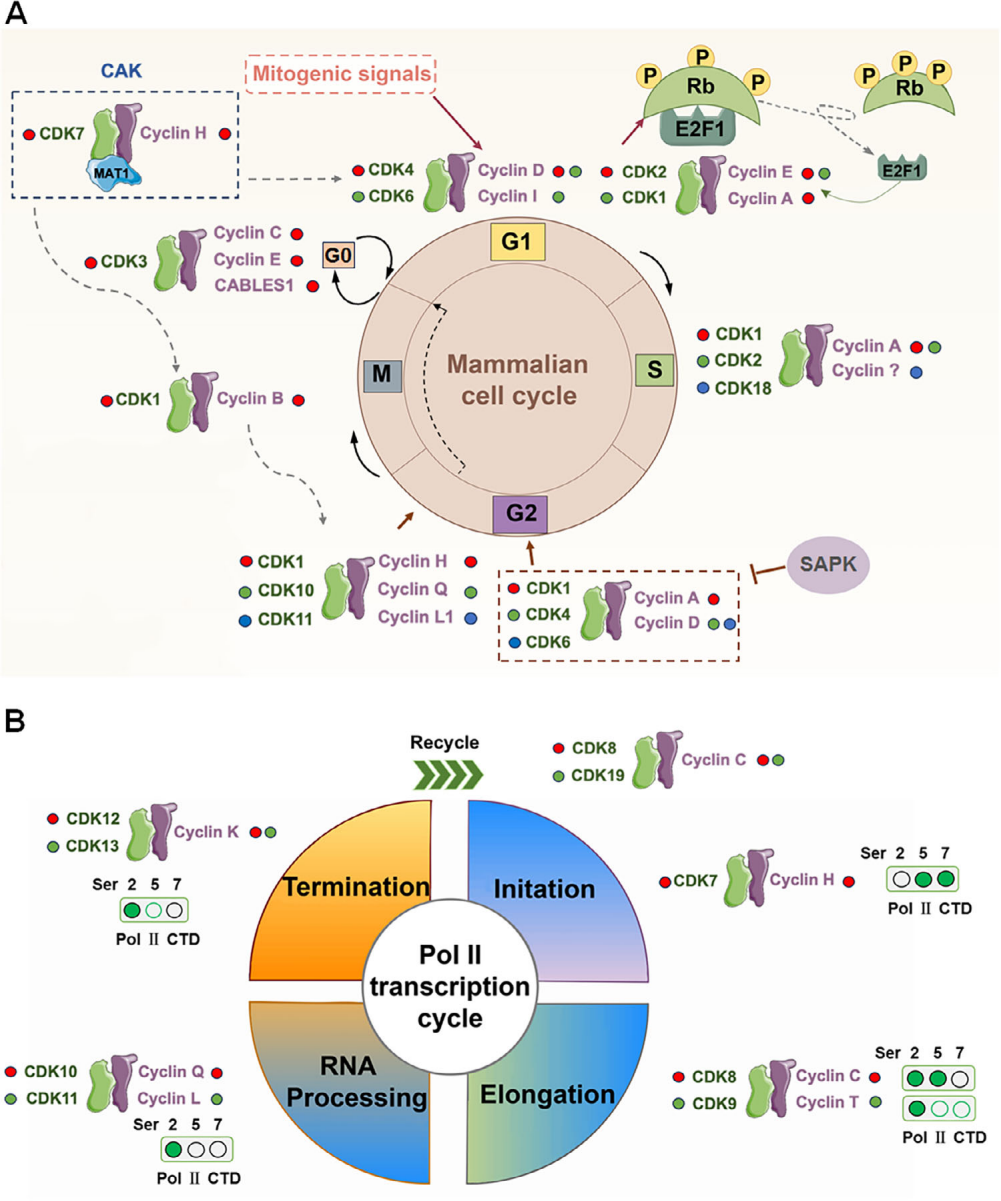
Function of CDKs and cyclins in cell cycle progression and Pol II transcription cycle. (A) Phase‐specific CDK/cyclin complexes enforce the temporal order of cell cycle events. Each CDK and its cyclin partner are marked by circles with the same color. *Abbreviations*: CAK, CDK‐activating kinase complex; SAPK, stress‐activated protein kinase. The exact cyclin that may dimerize with CDK18 in the S phase remains unclear. Inhibition of CDK1 and CDK4/6 by SAPK in the G2 phase potentiates cell cycle exit after the S phase. Black arrows denote cell cycle progression; other arrows indicate regulatory events between connected objects. (B) Impacts of selective CDK/cyclin complexes on phosphorylation of RPB1 CTD within the Pol II holoenzyme are depicted as solid green circles (regulations with high confidence), hollow green circles (putative or indirect regulations) or hollow black circles (no regulations). Each CDK and its cyclin partner are marked by circles with the same color.

Cell cycle‐regulatory CDKs orchestrate the temporal order of cell cycle events especially DNA replication and mitotic progression (Figure 3A). Normally, S phase‐associated CDKs and M phase‐associated CDKs regulate different pools of substrates, and S phase‐associated CDKs are unable to drive mitosis. However, in case that protein phosphatase 1 was removed from the centrosome, S phase‐associated CDKs could take over M phase‐associated ones to transcend substrate discrimination [52].

CDK1 is essential for cell cycle progression [32]. It pairs with cyclin A to promote mitotic entry from late G2 and participates in checkpoint control, ensuring proper DNA replication and repair before entry into mitosis. CDK1 is also partnered with cyclin B to phosphorylate multiple substrates, which promote chromosome condensation, nuclear envelope breakdown, and spindle assembly. Mitochondria‐localized CDK1/cyclin B1 phosphorylates various Complex I subunits of the electron transport chain, thereby enhancing mitochondrial respiration and ATP production to fuel energetic G2/M transition [53]. CDK1/cyclin B1 also regulates mitochondrial homeostasis by phosphorylating the mitochondrial fission regulator DRP1/DNM1L during mitotic progression [54]. In FLT3‐internal tandem duplication (FLT3‐ITD)‐positive acute myeloid leukemia, stabilized CDK1/cyclin B1 confers resistance to tyrosine kinase inhibitors [55]. Additionally, CDK1 is active during G1/S transition and functions redundantly with CDC7 in potentiating S phase entry [56]. CDK1 is able to compensate for persistent loss of CDK2 in Cdk2^−/−^ mice [32, 57]. Beyond cell cycle control, proficient CDK1 activity promotes epithelial–mesenchymal transition of triple‐negative breast cancer (TNBC) and adrenocortical carcinoma via downstream effectors including TWIST1 [58, 59]. However, excessive activation of CDK1 during hyperthermic intraperitoneal chemotherapy synergizes with WEE1 inhibition to suppress ovarian cancer [60]. A detailed summary of CDK1 biology in cancer can be found in the literature [61, 62].

CDK2 is another core cell cycle regulator that primarily dimerizes with cyclin E, cyclin A, cyclin B3, as well as cyclin J. CDK2/cyclin E complex favors the initiation of DNA replication and cell cycle transition from the late G1 phase to the S phase. CDK2/cyclin A complex coordinates the G1/S and G2/M transitions by phosphorylating key proteins in DNA replication, chromatin condensation and centrosome maturation [63, 64]. Oncogenic activation of CDK2 induces replication stress and DNA damage, which contributes to genomic instability and tumorigenesis [64]. In cancers with CCNE1 amplification, inhibition of CDK2 induces hypo‐phosphorylation of retinoblastoma protein (pRb) and therapy‐induced senescence [65], while inhibition of the PKMYT1 (protein kinase, membrane‐associated tyrosine/threonine 1), a negative regulator of CDK1, elicits synthetic lethal activation of CDK1 and premature mitosis [66]. In breast cancers, inhibition of CDK2 helps to retard and overcome acquired resistance to CDK4/6 inhibitors [65]. Conversely, CDK4/6 activity has been shown to enforce cellular adaptation to CDK2 inhibitors and sustain proliferative program [67]. Beyond cell cycle, CDK2 has been implicated in phosphorylating and activating various hormone receptors [68, 69, 70], and modulating several differentiation programs toward neuron and muscle. CDK2 activity also contributes to E2 promoter binding factor (E2F)‐mediated expression of DNMT1 whose activation depresses endogenous retroviral RNA and triggers Type I interferon (IFN) response [71]. Stimulation of IFN response by CDK2 inhibition subsequently promotes anticancer immunity by enhancing tumor antigen presentation and cytotoxic T‐cell infiltration. In addition, CDK2/cyclin J has been shown to suppress glycolysis and mitochondrial ROS production through phosphorylation of FOXK1 and DRP1/DNM1L in macrophages [72]. As a result, CDK2/cyclin J reduces innate immune response and metabolism in macrophages, promoting anticancer immunity. To date, several selective CDK2 inhibitors have been tested clinically and have shown early antitumor activity in cases with cyclin E overexpression or CCNE1/2 amplification [73]; representative ongoing clinical trials include those for INCB123667 (active trials: NCT07023627, NCT06909162, NCT07214779, NCT05238922), tagtociclib (active trials: NCT04553133, NCT05262400), AVZO‐021 (active trials: NCT05867251, NCT06998407), ECI830 (active trial: NCT06726148), AZD8421 (active trial: NCT06188520), BG‐68501 (active trial: NCT06257264), and INX‐315 (active trial: NCT05735080) (Table 1).

**TABLE 1:** Representative CDK/cyclin‐targeting agents in clinical trials.

Drug name	Targets	Mechanism	Tumor types	Trial ID	Phase	Status
INCB123667	CDK2/cyclin E1	Inhibitor	Platinum‐resistant ovarian cancer with cyclin E1 overexpression	NCT07023627	2	In progress
				NCT06909162	1	Completed
				NCT07214779	3	In progress
				NCT05238922	1	In progress
Tagtociclib	CDK2	Inhibitor	Advanced/metastatic small cell lung, breast and ovarian cancers	NCT04553133	2	In progress
			Breast cancer	NCT05262400	1/2	In progress
AVZO‐021	CDK2	Inhibitor	Advanced solid tumors, specifically, hormone receptor‐positive, HER2‐negative breast cancer, and cyclin E1 (CCNE1)‐altered malignancies	NCT05867251	1/2	In progress
ECI830	CDK2	Inhibitor	Advanced hormone receptor‐positive, HER2‐negative breast cancer or other advanced solid tumors harboring CCNE1 amplification	NCT06726148	1/2	In progress
AZD8421	CDK2	Inhibitor	Hormone receptor‐positive, HER2‐negative advanced breast cancer, and metastatic high‐grade serious ovarian cancer	NCT06188520	1/2	In progress
BG‐68501	CDK2	Inhibitor	Advanced solid tumors	NCT06257264	1	In progress
INX‐315	CDK2	Inhibitor	Hormone receptor‐positive, HER2‐negative breast cancer and cyclin E1 (CCNE1)‐altered malignancies	NCT05735080	1/2	In progress
Atirmociclib	CDK4	Inhibitor	Breast cancer	NCT06760637	3	In progress
			Advanced or metastatic breast cancer	NCT06105632	2	Completed
			Healthy participants	NCT07215078	1	Completed
			Advanced/metastatic estrogen receptor‐positive, HER2‐negative breast cancer	NCT05508906	1	In progress
			Metastatic breast cancer	NCT03424005	1/2	In progress
			Estrogen receptor‐positive, HER2‐negative advanced breast cancer	NCT07427394	2	In progress
BGB‐43395	CDK4	Inhibitor	Metastatic hormone receptor‐positive, HER2‐negative breast cancer, and other advanced solid tumors	NCT06120283	1	In progress
			Advanced/metastatic hormone receptor‐positive, HER2‐negative breast cancer	NCT07492641	1	In progress
			Advanced/metastatic hormone receptor‐positive, HER2‐negative breast cancer, and other solid tumors	NCT06253195	1	Completed
			Hormone receptor‐positive, HER2‐negative metastatic breast cancer	NCT06756932	1	In progress
SY‐5609	CDK7	Inhibitor	Advanced solid tumors (hormone receptor‐positive, HER2‐negative breast cancer, and pancreatic ductal adenocarcinoma)	NCT04247126	1	Completed
			Metastatic colorectal cancer	NCT04929223	1	In progress
Samuraciclib	CDK7	Inhibitor	Breast cancer	NCT04802759	1/2	In progress
			Estrogen receptor‐positive, HER2‐negative advanced or metastatic breast cancer	NCT06125522	1/2	In progress
			TNBC, castration‐resistant prostate cancer, and hormone receptor‐positive, HER2‐negative breast cancer	NCT03363893	1/2	Completed
			Metastatic or locally advanced hormone receptor‐positive, HER2‐negative breast cancer	NCT05963997	1/2	Completed
			Metastatic or locally advanced hormone receptor‐positive, HER2‐negative breast cancer	NCT05963984	2	Completed
Romaciclib	CDK8/19	Inhibitor	Acute myeloid leukemia	NCT06191263	2	Completed
			Intermediate or high‐risk, primary or secondary myelofibrosis	NCT06397313	2	Completed
			Lower‐risk myelodysplastic neoplasms	NCT06243458	2	Completed
Enitociclib	CDK9	Inhibitor	Solid tumors and aggressive non‐Hodgkin's lymphoma	NCT02635672	1	Completed
			Advanced hematological malignancies	NCT02745743	1	Completed
			Relapsed/refractory lymphoid malignancies	NCT05371054	1/2	Completed
SLS009	CDK9	Inhibitor	Relapsed/refractory hematologic malignancies and high‐risk newly diagnosed acute myeloid leukemia	NCT04588922	1/2	In progress
			Relapsed/refractory diffuse large B‐cell lymphoma	NCT06375733	1/2	In progress
			Relapsed/refractory peripheral T‐cell lymphoma	NCT05934513	1/2	Completed
Zotiraciclib	CDK9	Inhibitor	Recurrent malignant gliomas with IDH1 or IDH2 mutations	NCT05588141	1/2	In progress
			Elderly newly diagnosed or adult relapsed patients with anaplastic astrocytoma or glioblastoma	NCT03224104	1	Completed
			Advanced hematological malignancies	NCT01204164	1	Completed
			Relapsed/refractory chronic lymphocytic leukemia and small lymphocytic lymphoma	NCT01699152	1	Completed
NKT3964	CDK2	PROTAC	Advanced/metastatic solid tumors (with CCNE1 amplification)	NCT06586957	1	In progress
NKT5097	CDK2/CDK4	PROTAC	Advanced/metastatic solid tumors (emphasis on breast cancer and solid tumors with CCNE1 amplification)	NCT07029399	1	In progress
BTX‐9341	CDK4/CDK6	PROTAC	Advanced/metastatic hormone receptor‐positive, HER2‐negative breast cancer	NCT06515470	1	In progress
CT7439	Cyclin K CDK12/13	Molecular glue Inhibitor	Solid malignancies	NCT06600789	1/2	Completed

Data were retrieved from ClinicalTrials.gov by 2026‐May.

CDK3 is best known to interact with cyclin C, cyclin E1, and CABLES1 to promote cell cycle re‐entry from G0 phase (quiescent state), as well as G1/S transition [35, 74, 75, 76]. Protein tyrosine phosphatase nonreceptor Type 1 interacts with CDK3 and dephosphorylates its Tyr15 residue to enforce cell cycle progression in glioblastoma cells [77]. Activated CDK3 has been shown to phosphorylate pRb, TAZ (transcriptional coactivator with PDZ‐binding motif), transcription factors (TFs; e.g., JUN, ATF1, NFAT3), and transcriptional regulators including the NOTCH1 intracellular domain [78, 79, 80]. Ectopic CDK3 synergizes with MYC to confer oncogenic cell growth [81]. In colorectal cancer, CDK3 activates activating protein‐1 to induce epithelial–mesenchymal transition and enhance metastasis [82]. Interestingly, cancer genomics data indicate that CDK3 and CCNC are dysregulated by amplification and deletion, respectively (Figure 2A,B). The precise role of CDK3/cyclin C in cancer and the functional consequences of the above‐mentioned genomic defects are pending further clarification.

CDK4 and CDK6 selectively bind to D‐type cyclins (cyclin D1, D2, D3), and these complexes regulate the restriction point (R point) to control G1 to S phase transition [83, 84, 85]. CDK4/cyclin D and CDK6/cyclin D complexes initiate cell cycle progression through phosphorylation‐mediated inactivation of pRb and related pocket proteins p107/p130, thereby liberating E2F family TFs to activate S phase regulators including cyclin E1/E2. Subsequent assembly of CDK2/cyclin E complexes enforces S phase transition through coordinated substrate phosphorylation [86]. Loss of p53 does not change CDK4/6 activity but instead enhances CDK2‐dependent phosphorylation of p130 and enables cell cycle re‐entry under CDK4/6 inhibition. Simultaneous inhibition of CDK4/6 and CDK2 overcomes p53 loss and induces senescence [87]. Beyond cell cycle control, CDK4/6 couples proliferation with cell growth by binding to and phosphorylating TSC2 (Ser1452/Ser1217) [88]. Recent studies also reveal critical CDK4/6 activity beyond the R point in the interphase. Selective disruption of CDK4/6 activity in the S phase dampers CDK2 activity and pRb phosphorylation, resulting in cell cycle exit [89]. Similarly, inactivation of CDK4/6 in G2 phase (after DNA replication) by stress‐activated protein kinases (SAPKs) halts cell cycle progression [90]. CDK4/6 activity in G2‐arrested cells prevents stress‐induced endoreplication, a process generating polyploid cells through aberrant DNA replication without division. Intriguingly, CDK4/6 also exerts immune modulatory activities in cancer [91, 92, 93, 94, 95, 96]. CDK4/6 inhibition activates DNA damage and endogenous retrovirus in cancer cells, inducing genes involved in inflammatory and IFN responses, cGAS–STING1 signaling, antigen presentation, and immune cell interaction. CDK4/6 inhibitor treatment increases intratumoral CD8^+^ T cell infiltration while suppressing regulatory T cells, thereby enhancing anticancer immunity. Along with the successful clinical translation of CDK4/6 inhibitors, the expanding roles of CDK4/6 in cancer immunity support combination therapy with immune checkpoint inhibitors.

Apart from cell cycle regulation, CDK6 possesses noncanonical roles in regulating cancer metabolism and chromatin/transcription regulation. CDK6/cyclin D3 phosphorylates and inhibits 6‐phosphofructokinase (PFK1) and pyruvate kinase M2 (PKM2), redirecting glycolytic intermediates into the pentose phosphate and serine pathways to support cancer growth [97]. In BCR–ABL‐driven B cell acute lymphoid leukemia, CDK6‑defective cells showed an increased need for aerobic glycolysis, as both the kinase and chromatin‐regulatory activities of nuclear CDK6 are required for expression of genes involved in mitochondrial respiration [98]. CDK6 competes with nuclear respiratory factor 1 (NRF1) for binding at the promoters of metabolic gene targets. Moreover, CDK6 may work together with the avian erythroblastosis virus E26 oncogene homolog (ETS) proteins to maintain the chromatin accessibility and transcription of DNMT3B, extending its impact on DNA methylation [99]. The C‐terminus of CDK6 is critical for its nuclear translocation; subsequently, deletion of this C‐terminus further reduces its chromatin occupancy, as determined by chromatin immunoprecipitation sequencing [100]. Nuclear CDK6 also exerts kinase‐independent function through interacting with JUN to induce the proangiogenic factor VEGFA [101]. Handschick et al. revealed that TAK1 and mitogen‐activated protein kinase (MAPK/p38) promote CDK6 nuclear translocation and chromatin association upon IL‐1 stimulation, and that nuclear CDK6 further interacts with nuclear factor kappa‐light‐chain‐enhancer of activated B cells (NF‐κB/p65) and occupies enhancers and promoters of many IL‐1 target genes [102]. These findings support that CDK6 can be recruited to chromatin and regulates gene expression through both kinase‐dependent and ‐independent mechanisms.

The field of CDK4/6 inhibitors is indeed progressing at a remarkable pace. Regulatory approvals have been granted for the clinical use of palbociclib, ribociclib, abemaciclib, dalpiciclib, tibremciclib, lerociclib, fovinaciclib, and trilaciclib for targeted therapies, particularly against hormone receptor‐positive, HER2‐negative breast cancer (Figure S1). Remarkably, a CDK2/4/6 inhibitor culmerciclib and a CDK2/4/6/9 inhibitor bireociclib have also achieved translational success for similar indications (Figure S1) [11]. Furthermore, CDK4‐selective inhibitors represent a new frontier; leading candidates include atirmociclib (active trials: NCT06760637, NCT06105632, NCT07215078, NCT05508906, NCT03424005, NCT07427394) and BGB‐43395 (active trials: NCT06253195, NCT06120283, NCT07492641, NCT06756932) (Table 1).

### 4.2 Functions of Transcription‐Associated CDKs and the Associated Cyclins in Cancer

Transcription‐associated CDKs modulate gene transcription and transcription‐coupled RNA processing via phosphorylation of the YSPTSPS hepta‐repeats in the carboxy‐terminal domain (CTD) of DNA‐directed RNA Polymerase II (Pol II) subunit RPB1 (Figure 2B). CDK7 exemplifies a dual‐function kinase with central roles in both cell‐cycle progression and gene transcription. It associates with cyclin H and MAT1 to form the CAK complex, where it serves as the catalytic subunit phosphorylating many other cell cycle‐regulatory CDKs at the T‐loop to drive mitotic progression [103]. Moreover, CDK7 phosphorylates CTD of the Pol II RPB1 and T‐loop of multiple transcriptional CDKs (e.g., CDK9 at Thr186, CDK12 at Thr893, and CDK13 at Thr871) to facilitate transcription [104, 105]. CDK7 overexpression is associated with multiple malignancies, including TNBC, lung cancer, and MYCN‐driven neuroblastoma [106, 107, 108, 109]. CDK7 kinase activity facilitates Pol II escape from the promoter and promotes release of transcription initiation factors and mediator [110]. Conversely, CDK7 inhibition causes Pol II retention at promoters and further disrupts transcriptional homeostasis, super‐enhancer network, and genomic stability, thereby triggering antitumor immunity and attenuating therapeutic resistance [111, 112]. However, studies from CDK7‐selective inhibitors such like YKL‐5‐124 suggested that on‐target inhibition of CDK7 activity exerts a more pronounced inhibitory impacts on E2F expression program rather than Pol II CTD phosphorylation under the experimental conditions [113]. In addition, CDK7‐selective degraders (i.e., JWZ‐5‐13) demonstrated moderate antiproliferation efficacies despite robust depletion of CDK7, MAT1, and cyclin H [114]. Hence, many of the functional impacts by the previously reported CDK7 inhibitors (e.g., THZ1) may result from the off‐target inhibition of other CDKs, especially CDK12/13. Notably, CDK7 inhibitors are now entering advanced clinical trials, with encouraging results emerging from SY‐5609 (active trial: NCT04929223; completed trial: NCT04247126) and samuraciclib (active trials: NCT04802759, NCT06125522; completed trials: NCT03363893, NCT05963997, NCT05963984) evaluations (Table 1) [115]. Evolving insights into CDK7 biology reinforce the importance of selectivity in drugs aimed at targeting CDK7 for cancer treatment.

CDK8 and its closely related paralog CDK19 are alternative components of the kinase module and regulatory subunits within the mediator complex. Cyclin C and MED12 are needed for the activation of CDK8/19, while MED13 attaches the preassembled kinase module to the core mediator [116]. CDK8 phosphorylates hepta‐repeats of RPB1 CTD at Ser2 and Ser5 in vivo and in vitro [117], thereby influencing Pol II activity. Additional representative substrates include other mediator subunits and TFs including E2F1, signal transducer and activator of transcription proteins, and SMAD proteins [116]. Transcriptome analysis revealed that CDK8 kinase activity is essential for both basal and hypoxia‐induced expression of many glycolytic genes, including SLC2A1/3, HK1, ENO1/2, and PKM in colon cancer cells [118]. Inhibition of CDK8 reduces glucose uptake and glycolysis and sensitizes cancer cells to glycolysis inhibitor 2‐deoxy‐d‐glucose. Metabolomics revealed that CDK8/CDK19 may act as context‐specific and cell type‐specific regulators of the core metabolic pathways [119]. Inhibition of CDK8/CDK19 kinase activity rewires lipid metabolism in cells with hyperactive IFN gamma (IFNγ) signaling, and upregulates lipids with anti‐inflammatory activities. CDK8/19 may exert distinct regulatory impacts on genes based on transcriptional stages [116]. CDK8/19 likely have a negative regulatory impact on super‐enhancer‐driven transcription, and their inhibition in leukemia cells further increases the expression of super‐enhancer‐associated genes [120]. In hematopoietic stem cells and acute myeloid leukemia, CDK19 suppresses p53‐dependent expression of p21^Cip1^ and promotes cell proliferation [121]. CDK19 demonstrates better efficacy on proliferation of hematopoietic stem cells than CDK8. Codepletion of CDK8/19 impaired transcriptional activation of genes in response to various stimuli, for example, phorbol 12‐myristate 13‐acetate (PMA) and tumor necrosis factor [116]. Currently, a small molecule inhibitor romaciclib (SEL120), which targets the CDK8/19‐mediator complex, is being tested in clinical trials against various hematologic malignancies (active trials: NCT06191263, NCT06397313, NCT06243458) (Table 1). More studies are needed to explore the function and druggability of CDK8/19 in cancer.

CDK9 binds cyclin T (T1, T2a, and T2b) or cyclin K to form functional complexes mediating transcription elongation, mRNA maturation, and diverse physiological processes [122]. The CDK9/cyclin T complex constitutes the positive transcription elongation factor b (P‐TEFb), which phosphorylates Pol II RPB1 CTD to drive transcription elongation [123]. CDK9 contains an ATP‐binding site and a T‐loop with Thr186 residue, which must be phosphorylated by CAK to activate P‐TEFb. Guided by BRD4, AFF1, or AFF4 proteins, P‐TEFb localizes to gene promoters and phosphorylates RPB1 CTD at Ser2 and TFs (e.g., androgen receptor at Ser81) [124, 125]. This interaction promotes Pol II pause release and productive transcription elongation [126]. Disruption of CDK9 function not only impairs transcription elongation but also compromises RNA splicing and epigenetic silencing of endogenous retroviruses [127, 128]. Hence, CDK9 has emerged as a validated therapeutic target in transcriptionally addicted cancers [129, 130]. Moreover, targeting CDK9 blocks the transcriptional adaption to therapy and induces a metabolic switch. In leukemia cells driven by FLT3‐ITD, concurrent CDK9 depletion and FLT3 inhibition triggered synthetic lethality via attenuating purine synthesis and mitochondrial oxidative phosphorylation [131]. In prostate cancer, CDK9 inhibition suppresses oxidative phosphorylation and ATP production, causes acyl‐carnitine accumulation, and shifts cellular metabolism toward fatty acid oxidation dependence [132]. CDK9‐selective inhibitors are under clinical evaluation, including enitociclib (completed trials: NCT02635672, NCT02745743, NCT05371054), SLS009 (active trials: NCT04588922, NCT06375733, NCT05934513), and Zotiraciclib (active trial: NCT05588141; completed trials: NCT03224104, NCT01204164, NCT01699152) (Table 1) [133]. Remarkably, each of these agents has received United States Food and Drug Administration Orphan Drug Designation for non‐Hodgkin lymphoma, acute myeloid leukemia, and glioblastoma, respectively. Of note, two isoforms of CDK9 proteins are generated through alternative promoter usage and are evident in many cells [134]. The 42‐kDa short isoform of CDK9 is the predominantly expressed form and executes the general transcriptional functions of CDK9. The long isoform of CDK9 (55 kDa) contains an additional 117‐amino acid N‐terminal extension compared with the short isoform, while sharing identical sequences in the remaining regions. This long isoform was reported to localize predominantly to the nucleolus [135]. While regulation and precise contributions of long CDK9 isoform to human cancer remain enigmatic, emerging evidence implicates its role in DNA repair through Ku70 association and CDC23 (Ser588) phosphorylation [136, 137]. Selective depletion of long CDK9 isoform triggers a marked impairment of DNA end‐resection in response to camptothecin (DNA Topoisomerase I inhibitor) treatment and sensitizes cells to poly (ADP‐ribose) polymerase (PARP) inhibition [137]. Current CDK9‐targeting agents inhibit both isoforms, leaving the therapeutic potential of the long CDK9 isoform inadequately explored.

CDK10 functions with its partner cyclin Q (previously known as cyclin M) to form a kinase complex whose substrates include Pol II (RPB1 CTD Ser2), MEPCE, U2AF2, SRRT, NPM1, HSF1, MYC, pRb, ETS2, and PKN2 [138, 139, 140]. CDK10 phosphorylation at Thr196 within T‐loop is dispensable for CDK10/cyclin Q complex formation but important for its kinase activity. CDK1/cyclin B1 and CDK5/CDK5R are able to phosphorylate CDK10 at Thr49, Thr172, and Thr179 sites, suggestive of putative crosstalk between these CDKs. Cellular functions of CDK10 have been implicated in positive regulation of the G2/M phase, transcription, splicing, and actin dynamics [138, 140, 141, 142]. CDK10 is a candidate of tumor suppressor in breast cancer, liver cancer, biliary tract cancer, thyroid cancer, lung cancer, and glioma [143, 144, 145, 146, 147]. Reduced CDK10 expression confers resistance to endocrine therapy and enhances lymph node metastasis of breast cancer [148, 149, 150].

CDK11A and CDK11B are two homologous genes sharing over 99% protein sequence identity. Both of them pair with cyclin L and jointly promote assembly of the Pol II/mediator complex, gene transcription and RNA splicing. Multiple CDK11 isoforms (e.g., CDK11^p110^, CDK11^p58^) have been described and discussed [151, 152, 153, 154, 155], though their precise functions need further clarification [156, 157]. CDK11 has been shown to maintain centromeric transcription and expression of replication‐dependent histone genes [158, 159] and to enforce both steady‐state and induced transcriptional responses by controlling Pol II chromatin occupancy and nascent RNA synthesis through a Pol II pausing‐to‐elongation checkpoint upstream of CDK9 [160]. T‐loop phosphorylation of CDK11 at Thr595 (potentially by CDK7) is essential for its active complex with cyclin L and SAP30BP, which regulates spliceosome activity on chromatin in gene bodies [161]. Remarkably, CDK11 is also recognized as a “splicing‐associated CDK” that interacts with and phosphorylates various splicing factors (e.g., SF3B1); its impairment arrests the spliceosome at an intermediate complex B state, causing RNA splicing defects such as intron retention [162, 163, 164]. SCF^FBXW7^ controls ubiquitination and proteasomal degradation of cyclin L1 whose aberrant accumulation shortens G2/M phase and sensitizes the cell to CDK11 inhibition [165]. Phenotypically, CDK11 is important for the growth of liposarcoma, osteosarcoma, esophageal squamous cell carcinoma, melanoma, and neuroblastoma cells [166, 167, 168, 169, 170, 171]. Among reported CDK11 inhibitors (e.g., OTS964, OTS514, ZNL‐05‐044, compound 37) [172, 173, 174, 175, 176], dual CDK11/PBK inhibitors demonstrated promising preclinical antitumor activities.

CDK12 and CDK13 form complexes with cyclin K to phosphorylate RPB1 CTD Ser2, thereby coordinating Pol II elongation and cotranscriptional RNA processing by recruiting splicing and polyadenylation factors. Phosphorylation of CDK12 T‐loop at Thr893 by CAK is required for the full activation of CDK12/cyclin K complex [25]. CDK13 also interacts with cyclin T1 to phosphorylate ZC3H14, a component of polyA exosome targeting complex that targets prematurely terminated RNAs for clearance [49]. Distinct from the well‐characterized CDK9‐containing complexes in transcriptional control, CDK12 demonstrates unique substrate specificity by additionally targeting LEO1 (Ser608) within the PAF1 (Pol II‐associated factor 1) complex, a modification that enhances transcriptional processivity by stabilizing elongation complexes [177]. While individual inhibition of CDK12 or CDK13 induces DNA damage response (DDR) pathway activation without substantial cytotoxicity, their coinhibition disrupts transcriptional homeostasis through three convergent mechanisms: premature cleavage and polyadenylation, intragenic transcription termination, and widespread splicing dysregulation. This combinatorial effect reveals a critical functional redundancy between CDK12 and CDK13 in maintaining transcriptional fidelity [178, 179, 180]. CDK12 orchestrates transcription elongation and splicing fidelity of homologous recombination (HR) repair genes, notably BRCA1/BRCA2 and ATR, through suppression of intronic polyadenylation. Genetic ablation of CDK12 disrupts this transcriptional regulation program, resulting in impaired HR‐mediated DNA repair capacity, accumulation of chromosomal instability, and selective vulnerability to PARP inhibitors and platinum‐based chemotherapeutics [181, 182, 183, 184]. Preclinical studies demonstrate that CDK12/13 inhibitors recapitulate this HR deficiency phenotype and synergize with PARP inhibitors [183]. Genetic or pharmacological depletion of CDK12/13 also activates the STING1 signaling pathway and synergizes with immunotherapy in syngeneic tumor models [185]. These kinases mediate essential functional crosstalk between transcription elongation machinery and nucleic acid surveillance pathways. Specifically, while CDK12 coordinates HR‐mediated genome integrity through regulation of DDR genes, CDK13 maintains transcriptome fidelity by activating nuclear RNA surveillance and modulating splicing factor recruitment [49]. This functional divergence establishes a synthetic lethal paradigm wherein concurrent inhibition of both kinases unveils a therapeutic vulnerability in HR‐deficient malignancies. The mechanism of action involves targeted disruption of transcription‐coupled regulatory networks that maintain proteostasis under replicative stress, effectively exploiting cancer‐specific dependencies in DNA repair‐deficient tumors [186, 187]. In parallel, CDK12 has important impacts on cancer metabolism. For example, CDK12‐deficient castration‐resistant prostate cancer showed elevated mitochondrial respiration and ATP synthesis, and reduced expression of ACSL4 to escape ferroptosis [188]. Overexpression of CDK12 promotes breast cancer development and progression, in part, through enforcing expression of metabolic genes involving folate cycle and serine synthesis pathway [189]. Excessive CDK12 reprograms the serine‐glycine‐one‐carbon metabolism in breast cancer and confers positive response to methotrexate‐based chemotherapy by targeting dihydrofolate reductase activity. Importantly, disease‐ and context‐dependent functions of CDK12 and/or CDK13 warrant further clarification. Some human tumors harboring loss‐of‐function mutations in CDK12 have been shown to exhibit a BRCAness phenotype due to increased intronic polyadenylation of DDR genes, suggesting enhanced responsiveness to PARP inhibitors and immune therapy [182]. Separately, biallelic loss of CDK12 defines an immunogenic molecular subtype of metastatic castration‑resistant prostate cancer (i.e., the CDK12 loss and tandem duplicator phenotype), which harbors higher levels of neoantigens and tumor‐infiltrating lymphocytes and may be targetable by immune checkpoint inhibitors [48]. However, these CDK12‐loss, advanced prostate cancers lack mutational signatures of HR deficiency, contrasting with the phenotypes induced by acute CDK12 perturbation in most cell‐based assays. Therefore, it is important to distinguish between disease/context‐associated tumor biology shaped by genetic loss of CDK12 and the consequences of acute pharmacological CDK12 inhibition.

### 4.3 Functions of Atypical CDKs and the Associated Cyclins in Cancer

CDK5, CDK14, CDK15, CDK16, CDK17, and CDK18 all belong to atypical CDKs that are understudied in cancer [190]. CDK5 is well known for its postmitotic functions in the central nervous system [191, 192], while knowledge of its roles in either cell cycle or cancer remains less studied. In postmitotic cells, CDK5 is activated by cyclin I, CDK5R2 (p39 and its cleaved product p29), and CDK5R1 (p35 and its cleaved product p25) [193], while cyclin D and cyclin E counteract the interactions between CDK5 and its activators [194, 195]. Expression of the aberrant CDK5 activator p25 in murine β‐islet cells drives pancreatic neuroendocrine tumor development (approximately half of which are potential insulinomas) [196]. Selectively inhibiting CDK5/p25 complex while preserving CDK5/p35 function by a 24‐amino‐acid peptide (TP5) exerts antiproliferative activity in colorectal cancer models [197], suggesting a CDK5/p25‐biased oncogenic addiction. Of note, Xiao et al. identified CDK5 as a host target of the human papillomavirus (HPV) oncoprotein E6 [198]. The CDK5 inhibitor CP681301 interferes with the CDK5/E6 complex and counteracts the malignant phenotypes of HPV‐positive cancer cells, establishing CDK5 as a potential drug target for HPV‐associated diseases. CDK5 modulates programmed death‐ligand 1 (PD‐L1) expression in a context‐dependent manner. In medulloblastoma, CDK5 fosters the IFNγ‐induced upregulation of PD‐L1 via suppressing PD‐L1 transcriptional repressors IRF2 and IRF2BP2 [199]. In lung cancer, CDK5 maintains PD‐L1 expression via the ubiquitination–proteasome pathway [200, 201], whereas in hepatocellular carcinoma (HCC), CDK5 phosphorylates PD‐L1 at Thr290 and promotes its degradation via chaperon‐mediated autophagy [202]. CDK5 expression and activity are regulated by tumor microenvironment cues. CDK5 expression is induced by high glucose, but is repressed by dexmedetomidine in kidney cancer cells [203]. Its activity is stimulated by GDNF‐induced RET signaling in medullary thyroid cancer, but is compromised by DYRK1A, which is associated with differentiation signals in glioblastoma cells [204, 205]. During mitotic progression, CDK5 is phosphorylated at Ser159 and forms a functional complex with cyclin B1 to protect mitotic fidelity. Loss of CDK5 in the mitotic cells causes hyperstable spindles, lagging chromosomes and micronuclei [206]. CDK5 also interacts with p21^Cip1^ and reduces its stability [207]. As a kinase, CDK5 phosphorylates serine or threonine residues within the consensus motif S/TPXH/K/R of protein substrates. In cancer, CDK5 exhibits glucose‐associated functions: upon glucose starvation, it phosphorylates NOXA (phorbol‐12‐myristate‐13‐acetate‐induced protein 1) at Ser13 to antagonize apoptosis [208]; under high glucose condition, it interacts with and phosphorylates DRP1 to facilitate epithelial–mesenchymal transition [203]. CDK5 also indirectly regulates glutamine metabolism via phosphorylating EZH2, thereby promoting polycomb repressive complex 2‐dependent control of glutamine transporters and tricarboxylic acid cycle enzymes in colorectal cancer cells [209]. Additional CDK5 substrates include ATM (Ser794), vimentin (Ser56), focal adhesion kinase (FAK; Ser732), MCM2 (Ser27), PRMT1 (Ser307), STAT3 (Ser727), PIKE isoform A (Ser279), TRIM59 (Ser308), MYC (Ser62), TPX2 (Ser486), and ADD1 (Thr724) [210, 211, 212, 213, 214, 215, 216, 217, 218, 219, 220]. By catalyzing the phosphorylation of key signaling molecules, CDK5 potentially serves as a signaling hub that integrates the Hippo, STAT3, mammalian target of rapamycin (mTOR), NOTCH, and Wnt pathways [220, 221, 222, 223]. For example, CDK5 cooperates with LIMK1 to phosphorylate β‐catenin at Ser191, promoting its nuclear translocation and the activation of key Wnt target genes in cancer metastasis [224]. Consequently, CDK5 is able to promote cell growth, invasion, metastasis, and immune evasion, making it a potential target for cancer therapy [219, 225, 226, 227].

CDK14 and CDK15 are known as the PFTAIRE (CBH with a conserved motif of Pro–Phe–Thr–Ala–Ile–Arg–Glu) subclass of CDKs. Both CDK14 and CDK15 interact with cyclin Y and, to a lesser extent, with cyclin I [228]. CDK14 has a supportive role in lung development and repair [229], as well as in mitotic cell cycle and malignant progression of bone, brain, breast, esophageal, lung, and ovarian cancers [228, 230, 231, 232, 233, 234, 235, 236, 237, 238]. CDK14 ablation inhibits the proliferation of lung epithelial and vascular endothelial cells, inducing G2/M phase cell cycle arrest. Consequently, genetic knockout of CDK14 impairs pulmonary vascular endothelial cells and alveolar epithelial cells during mouse embryonic development, as well as lung repair following bleomycin‐ or lipopolysaccharide‐induced injury, potentially through crosstalk with the IFNγ–STAT1 pathway [229]. In lung cancer, the biogenic amine 5‐hydroxytryptamine has been shown to promote malignant phenotypes and metastasis through inducing CDK14 expression [233]. FMF‐04‐159‐2 is a potent and covalent inhibitor, which can be used as a tool compound for CDK14 research [236]. Wnt/β‐catenin signaling represents one of the key downstream pathways affected by CDK14 inhibition and mediates CDK14's function in sustaining proliferation, survival, invasion, and metastasis [228, 230, 231, 239, 240, 241]. CDK15 has been implicated in progression of breast and colon cancers [242, 243]. CDK15 enhances phosphorylation of survivin at Thr34 and PAK4 at Ser291, thereby conferring TRAIL (tumor necrosis factor‐related apoptosis‐inducing ligand) resistance, proliferation, and anchorage‐independent growth to cancer cells [242, 243]. Paradoxically, loss of CDK15 was also reported to enhance breast tumor cell invasion and metastasis [244].

CDK16 (PCTK1, PCTAIRE1), CDK17 (PCTK2, PCTAIRE2), and CDK18 (PCTK3, PCTAIRE3) form the PCTAIRE (CBH with a conserved motif of Pro–Cys–Thr–Ala–Ile–Arg–Glu) subfamily of CDKs, which remain inadequately evaluated to date. All three CDKs may partner with cyclin Y and cyclin I, whereas CDK16 additionally interacts with cyclin Y‐like 1 [228, 245, 246, 247]. Of note, CDK16 forms a complex with cyclin Y and 14‐3‐3 proteins rather than with cyclin Y alone [41, 248]. CDK16 binds the cyclin box of cyclin Y, while 14‐3‐3 provides additional contacts, including with the CDK16 activation segment. Cyclin Y also engages the CDK16 N‐terminal extension for full activation. CDK16 Ser119 resides within a binding motif for multiple upstream kinases, 14‐3‐3 protein, and cyclin Y‐like 1. The isolated CDK16 kinase domain is relatively unstable without its cyclin partner [249]. AMPK‐dependent phosphorylation of cyclin Y at Ser326 promotes its interaction with CDK16 and enhances autophagy [250]. Beyond cyclins, other key interactors of CDK16 include PRKAR1A (also known as KAP0), PLK1, AKT1, LKB1, and BRCA1 [251, 252, 253]. Phosphorylation by AKT1 enhances CDK16 stability, while LKB1 and BRCA1 may promote its degradation. Duan et al. reported that copper directly binds and activates CDK16, enhancing its interaction with JAK1 and driving JAK1‐dependent upregulation of MYC and cyclin D1 in TNBC [252]. Supporting its role as a mitotic kinase, CDK16 regulates spindle orientation in a kinase activity‐dependent manner [251]; its knockdown causes aberrant mitosis by impairing spindle assembly and chromosome segregation [253]. Defective CDK16 triggers G2/M arrest and apoptosis in TNBC and medulloblastoma cells [254, 255]. CDK16 abundance and its phosphorylation status fluctuate across the cell cycle, peaking at mitosis, accompanied by dynamic subcellular localization: at centrosomes during G2, at spindle poles upon mitotic entry, and at the midbody during cytokinesis [253]. In addition, CDK16 has been implicated in phosphorylation of p27^Kip1^, AAK1, p53, ARID1A, and protein regulator of cytokinesis 1 (PRC1) [256, 257]. CDK16 sustains the pRb phosphorylation at Ser780 and reduces p27^Kip1^ levels in RAS‐driven melanoma cells [258]. Similarly, CDK16 negatively regulates cell proliferation and apoptosis partially through promoting ubiquitination and proteasomal degradation of p27^Kip1^ in non‐small cell lung cancer [259]. In terms of therapeutic relevance, CDK16 promotes resistance to tumor necrosis factor family cytokines and antimitotic drugs including paclitaxel and nocodazole [253, 260]. CDK16‐positive breast cancer cells featured with a luminal progenitor cell‐like expression signature are associated with resistance to neoadjuvant chemotherapy [261]. To date, CDK16 knockdown has been shown to inhibit cell viability in colorectal cancer, melanoma, non‐small cell lung cancer, prostate cancer, breast cancer, osteosarcoma, and medulloblastoma. However, CDK16 may have lineage‐specific functions on inhibiting proliferation and stemness of colorectal cancer cells [262, 263]. Remarkably, CDK16 cocrystallizes with several chemical scaffolds, including the Indirubin derivative E804 and rebastinib, providing a foundation for developing more selective inhibitors [249]. CDK18 (PCTAIRE 3) emerges as a rate‐limiting regulator of ATR‐regulated DNA replication stress signaling [264, 265]. CDK18 deficiency reduces chromatin retention of RAD9 and RAD17 in response to replication stress, resulting in an increase in endogenous DNA damage and chromosomal abnormalities. Silencing of CDK18 is also able to impair cell cycle by arresting cells in early S phase, promote cell motility by actin reorganization, as well as reduce the ERK1/2 activity and confer PARP inhibitor sensitivity in various lineages [266, 267, 268]. CDK18 controls actin cytoskeleton dynamics by negatively regulating the FAK phosphorylation and the downstream RhoA/Rho‐associated kinase activity [266]. Overexpression of CDK18 promotes filopodia formation while reducing cell adhesion in Hela cells. Several review articles provide additional structural and functional information of the PCTAIRE CDKs [269, 270]. Cancer‐associated functions for CDK17 (PCTAIRE 2) remain poorly reported.

CDK20 (also known as CCRK) enforces cell proliferation by partnering up with cyclin H and exhibits context‐dependent CAK activity toward CDK2 [271, 272, 273]. CDK20 also interacts with FGFR3, although the functional consequences of this interaction remain unknown [274]. CDK20 transcription can be induced by IL‐6, STAT3, the androgen receptor, and a splice variant of NCOR2 (BQ323636.1) [275, 276, 277]. Accumulating studies reveal a dual role of CDK20 in both cancer cell‐intrinsic processes and immune regulation. Silencing of CDK20 induces G1 phase arrest in various cancer cells [278]. Elevated CDK20 expression is associated with reduced patient survival and poor immunotherapy responsiveness in HCC [279]. Hepatic CDK20 promotes obesity‐associated lipid accumulation, glucose intolerance, insulin resistance, and HCC development through glycogen synthase kinase 3 beta (GSK3β)‐mediated mTORC1 activation in male mice fed with a high‐fat, high‐carbohydrate diet [277]. Concurrently, hepatic CDK20 stimulates mTORC1‐dependent granulocyte colony stimulating factor to recruit myeloid‐derived suppressor cells (MDSCs), thereby establishing a protumorigenic microenvironment. CDK20 overexpression promotes HCC cell proliferation, clonogenicity, and elevation of EGFR and nonphospho‐β‐catenin, all of which can be reversed by Bufalin, a bioactive component from traditional Chinese medicine with antitumor potential [280]. Pharmacological inhibition of CDK20 with a small molecule inhibitor ISM042‐2‐048 demonstrates selective antiproliferation activity against an HCC cell line Huh7 [281]. CDK20 inhibition has also been shown to attenuate immune evasion of liver cancer cells by lowering IL‐6 expression and restraining IL‐6‐dependent MDSCs [282]. Zhou et al. further showed that intratumoral injection of CDK20‐deficient MDSCs promotes tumor‐infiltrating CD8^+^ T cells and suppresses HCC tumorigenicity [279]. Mechanistically, CDK20 reinforces STAT3/E4BP4 signaling in MDSCs, conferring immunosuppressive activity by switching cytokine production from IL‐12 to IL‐10. CDK20 has also been implicated in phosphorylation of MAK‐related kinase/intestinal cell kinase and GSK3β, stabilization of NFE2 like BZIP TF 2 (NRF2) and Ku70, as well as activation of EZH2‐mediated transcription, the Hedgehog pathway, and the Wnt/β‐catenin signaling pathway [275, 282, 283, 284, 285, 286, 287].

In brief summary, current understanding of CDKs and cyclins highlights their importance in diverse aspects of cancer biology. Targeting CDK/cyclin proteins is likely to have broad impacts on cell cycle, transcription, DNA repair, DNA replication, RNA processing, signaling transduction, metabolism, and immune response. Nevertheless, functions of most atypical CDKs remain to be characterized further.

## 5 Targeted Protein Degradation Technologies for CDK/Cyclin Modulation

The functional prominence of CDK/cyclin proteins has driven extensive development of CDK/cyclin inhibitors for cancer intervention. In general, CDK inhibitors are classified into three primary categories based on their mechanism of action: ATP‐competitive inhibitors, covalent inhibitors, and allosteric inhibitors. The development of CDK inhibitors has demonstrated significant progress, with a number of therapeutic candidates targeting catalytic activity advancing through clinical trials and several CDK4/6‐selective or ‐biased agents receiving regulatory approval. Nevertheless, achieving target selectivity remains a great challenge in conventional inhibitor design, attributable to the intrinsic limitations including high sequence homology across the CDK family (particularly within ATP‐binding pockets).

**FIGURE 4:**
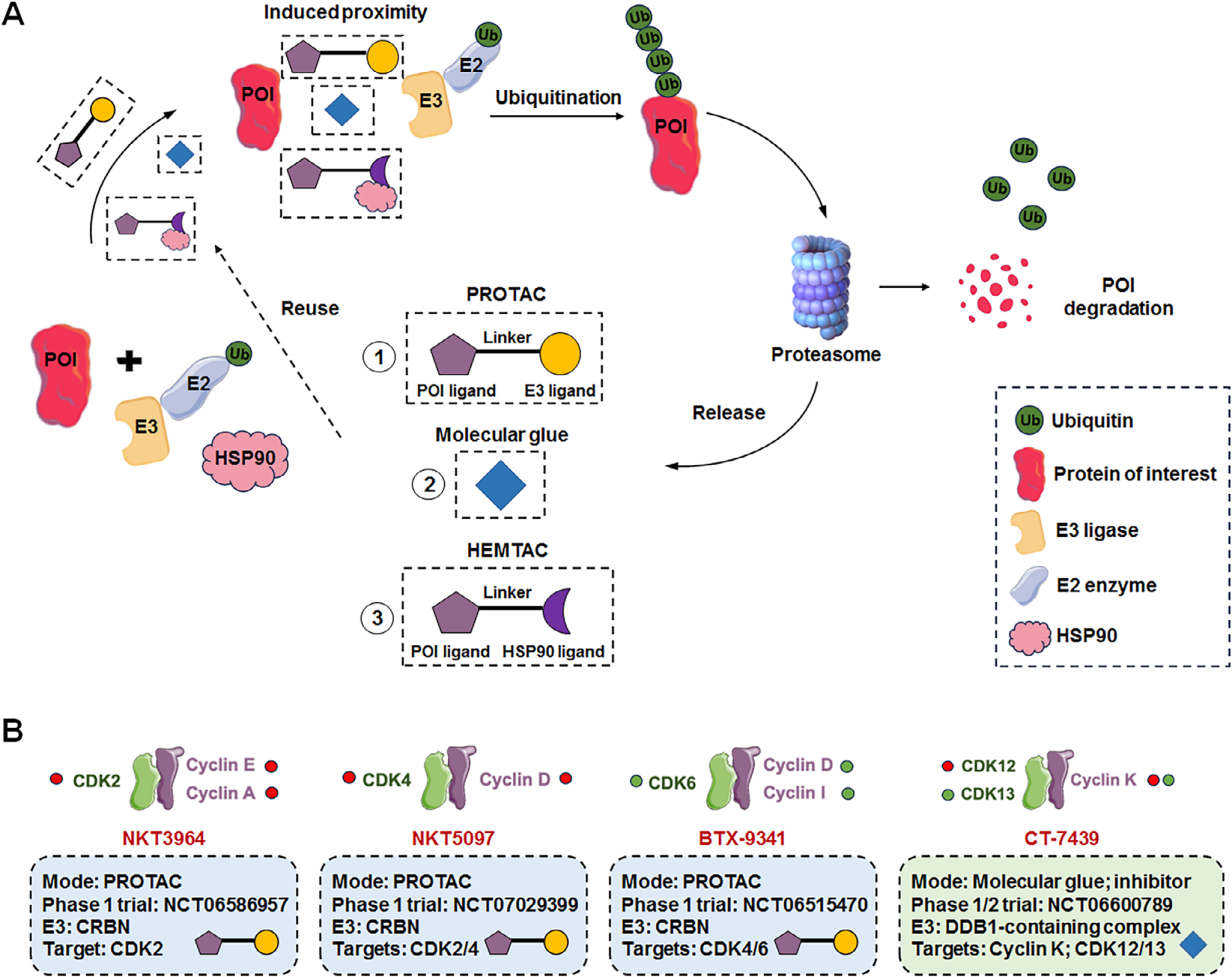
Working mechanisms of PROTACs, HEMTACs, and molecular glues, and information on CDK/cyclin‐targeting degraders under clinical investigation. (A) PROTAC induces proteasome‐dependent degradation of POI via formation of a POI–PROTAC–E3 ternary complex. HEMTAC recruits various E3 ubiquitin ligases via the adaptor protein HSP90 and subsequently forms a POI–HEMTAC–HSP90–E3 complex to induce targeted protein degradation. Molecular glue alters protein‐protein interaction surfaces between an E3 ligase complex and POI or a complex containing POI, thereby either creating or enhancing the interaction affinity to trigger the proximity‐induced protein degradation. Arrows denote individual working steps in the depicted process. (B) CDK/cyclin‐targeting degraders (PROTACs and a molecular glue) in active clinical trials.

**FIGURE 5:**
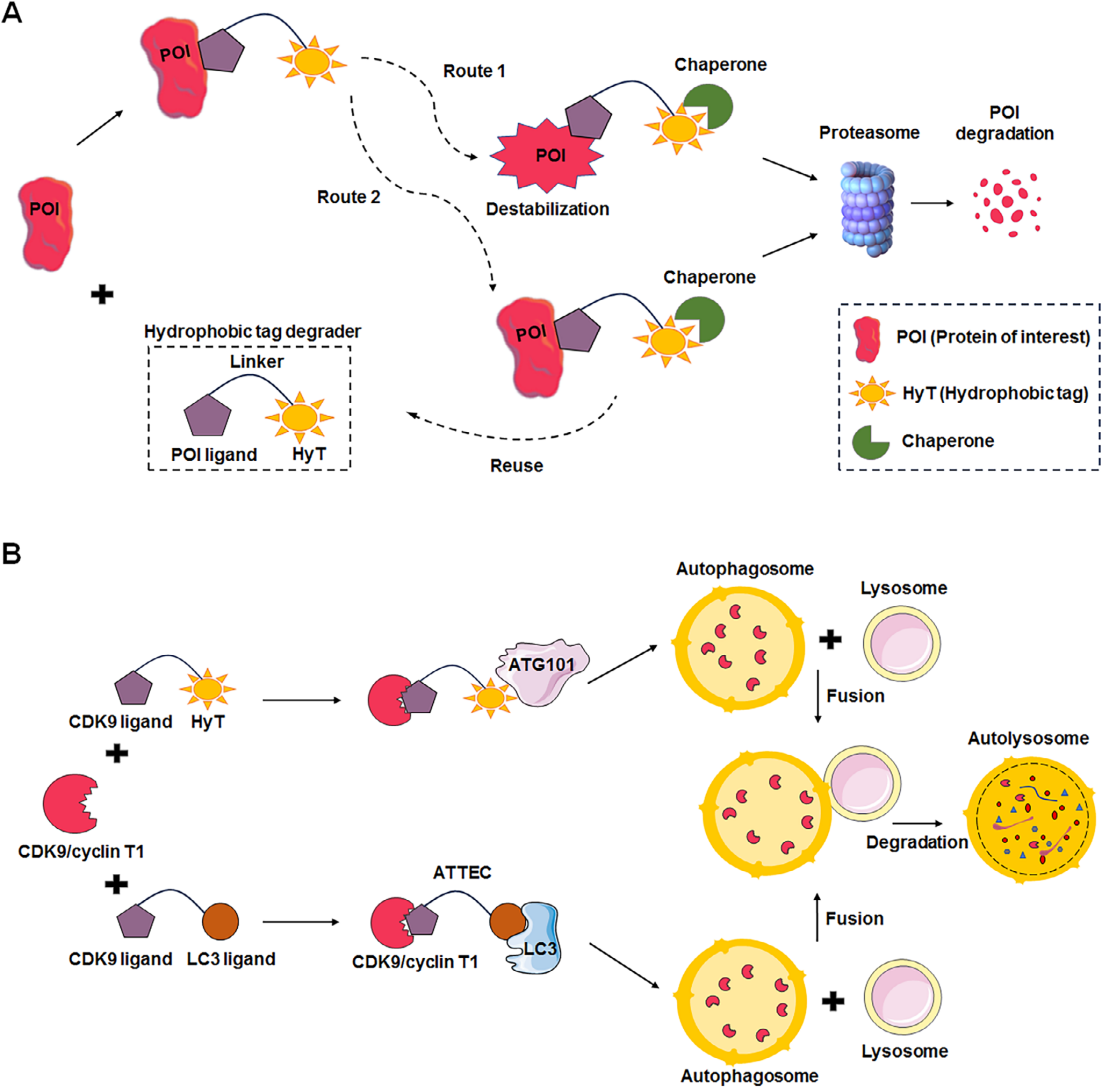
Working mechanisms of hydrophobic tag degraders and autophagy‐tethering compounds. (A) HyTs engage POI by either directly disrupting folding pathways to induce structural instability (Route 1), or mimicking partial denaturation to expose hydrophobic moieties on the protein surface (Route 2). In both cases, these actions generate molecular signals of abnormal folding or overwhelm chaperone‐mediated quality control, thereby activating the proteasomal degradation machinery to target POIs for elimination. Arrows denote individual working steps in the depicted process. (B) HyTs and ATTECs degrade POI via autophagosome–lysosome pathway. ATG101 and LC3 proteins are exploited as autophagosome receptors by the reported HyTs and ATTECs, respectively. The formation of a ternary complex involving CDK9/cyclin T1–HyT–ATG101 or CDK9/cyclin T1–ATTEC–LC3 facilitates engulfment of the complex into autophagosomes. CDK9/cyclin T1 is subsequently degraded following autophagosome–lysosome fusion. Arrows denote individual working steps in the depicted process.

The advent of targeted protein degradation technologies has revolutionized therapeutic approaches for CDK/cyclin intervention, shifting the paradigm from catalytic inhibition to protein elimination. This review outlines the technical schemes of targeted CDK/cyclin degradation, mainly including PROTAC, molecular glue, HEMTAC, HyT, and ATTEC (Figures 4 and 5).

### 5.1 PROTAC

PROTACs are bifunctional molecules composed of two key moieties: a ligand that selectively binds to a protein of interest (POI) and a ligand that engages an E3 ubiquitin ligase, connected by a chemical linker [288]. These molecules act by bridging the target protein and the E3 ligase to form a ternary complex, thereby facilitating ubiquitination of the POI and its proteasomal degradation (Figure 4A). Conventional “occupancy‐driven” inhibitors rely heavily on their affinity to effectively block target activity (e.g., by occupying the ATP‐binding pocket of CDKs), a mechanism that requires continuous maintenance of high plasma and intratumoral free drug concentrations (above the half‐maximal inhibitory concentration, IC_50_) to ensure sustained target occupancy and subsequent perturbation of enzymatic function. In contrast, PROTACs operate via an “event‐driven,” catalytic mechanism [289, 290]. The catalytic nature of PROTACs allows a single molecule to trigger multiple rounds of target degradation [291]. Consequently, only transient exposure (above the half‐maximal degradation concentration, DC_50_) within tumors is sufficient to initiate the degradation cascade, reducing the need for prolonged high systemic concentrations and potentially lowering off‐target toxicity. Furthermore, PROTAC‐induced degradation eliminates both the enzymatic and scaffolding functions of POI, thereby impacting pharmacodynamic readouts and pathological roles that extend beyond enzymatic activity [292, 293]. Recently, the estrogen receptor‐targeting PROTAC ARV‐471 (vepdegestrant) demonstrated positive clinical effects on metastatic breast cancer patients with ESR1 mutations in a Phase 3 VERITAC‐2 clinical trial (NCT05654623). Based on these results, it has received official approval for the treatment of adults with estrogen receptor‐positive/HER2‐negative, ESR1‐mutated advanced or metastatic breast cancer. Meanwhile, PROTACs targeting the androgen receptor (ARV‐110, ARV‐766), and Bruton's tyrosine kinase (NX‐2127) have also progressed to clinical evaluation in various cancers [294, 295]. Encouragingly, three CRBN‐recruiting PROTAC compounds targeting cell cycle‐regulatory CDKs have entered early‐phase clinical trials (Figure 4B and Table 1): NKT3964 targets CDK2 (active trial: NCT06586957); NKT5097 targets CDK2 and CDK4 (active trial: NCT07029399); and BTX‐9341 degrades CDK4 and CDK6 (active trial: NCT06515470).

The preclinical development of CDK‐targeting PROTACs has become a fast‐evolving cutting edge in pharmacological research. CDK PROTACs has demonstrated the advantages to circumvent resistance mechanisms associated with traditional ATP‐competitive inhibitors, and can provide mechanistic insights into context‐dependent CDK functionalities. While the developmental history and landscape of CDK PROTACs have been reviewed elsewhere [3, 296], we here highlight selective CDK PROTACs with disclosed structures that demonstrate both exceptional target discrimination and robust preclinical therapeutic efficacy (Figure S2).

#### 5.1.1 Selective PROTACs Targeting Cell Cycle‐Regulatory CDKs

The rational design of CDK4/6‐targeting PROTACs has predominantly employed structural conjugation of clinically validated CDK4/6 inhibitors with CRBN‐ or VHL‐recruiting E3 ligase ligands (e.g., thalidomide and pomalidomide) [297, 298, 299]. Notable examples include: BSJ‐03‐204 (derived from palbociclib), demonstrating dual degradation of CDK4/CDK6; BSJ‐04‐132 (ribociclib‐based), achieving CDK4‐selective depletion; and palbociclib‐derived BSJ‐03‐123 and CP‐10, exhibiting preferential CDK6 degradation at low nanomolar concentrations (Table 2 and Figure S2). Developments in CDK2‐selective PROTACs feature CPS2, PROTAC‐8, and CDK2 degrader 37 (Table 2 and Figure S2) [300, 301, 302]. CPS2, engineered through strategic fusion of a JNJ‐7706621 derivative (J2) with pomalidomide, demonstrated good degradation selectivity for CDK2 and AURKA, and showed promising antileukemic activity. PROTAC‐8 was developed based on the chimeric design of a CDK2 inhibitor AZD5438 and a VHL binding probe VH032. PROTAC‐8 displayed strong target specificity but only moderate degradation efficiency, urging further structural optimization. In addition, the anticancer potential of PROTAC‐8 has not been explored. CDK2 degrader 37 has recently been developed as a CRBN‐dependent PROTAC degrader of CDK2, showing favorable oral bioavailability and strong antitumor activity in tumor models with CCNE1 amplification. This compound exhibits low‐nanomolar antiproliferative activity (IC_50_ = 9 nM; DC_50_ = 13 nM) in MKN1 cells (with CCNE1 amplification), compared with micromolar activity in cells with diploid CCNE1 [301].

**TABLE 2:** Representative CDK/cyclin‐targeting degraders and relocalization agents.

Compound	Mechanism	Targets	Responsive cells	In vivo anticancer activity	E3 dependency	References
BSJ‐03‐204	PROTAC	CDK4 (DC_50_ < 100 nM) CDK6 (DC_50_ < 100 nM) in Jurkat cells with 4‐h treatment	Jurkat, mantle cell lymphoma cell lines	Not reported	CRBN	[299]
BSJ‐04‐132	PROTAC	CDK4 (DC_50_ ≈ 100 nM) in Jurkat cells with 4‐h treatment	Jurkat	Not reported	CRBN	[312]
BSJ‐03‐123	PROTAC	CDK6 (DC_50_ < 100 nM) in Jurkat cells with 4‐h treatment	MV‐4‐11, MOLM13, etc.	Not reported	CRBN	[299, 312]
CP‐10	PROTAC	CDK4 (DC_50_ > 100 nM) CDK6 (DC_50_ = 2.1 nM) in U251 cells with 24‐h treatment	U251, HL‐60, RPMI8226, MM.1S, Jeko‐1, etc.	Not reported	CRBN	[298]
CPS2	PROTAC	CDK2 (DC_50_ = 12 nM) AURKA (DC_50_ unclear) in NB4 cells with 48‐h treatment	NB4, U937, OCI‐AML2, OCI‐AML3, MV‐4‐11, K562, DOHH2, Ramos, Jurkat, MDA‐MB‐231, etc.	Not reported, except for good tolerability	CRBN	[300]
PROTAC‐8	PROTAC	CDK2 (DC_50_ ≈ 100 nM) in HEI‐OC1 cells with 24‐h treatment	HEI‐OC1	Not reported, except for protective effects against hearing loss in zebrafish	VHL	[302]
Degrader 37	PROTAC	CDK2 (DC_50_ = 13 nM) in MKN1 cells	MKN1, HCC1569	Yes, tumor‐inhibitory activity against HCC1569 xenograft model	CRBN	[301]
JWZ‐5‐13	PROTAC	CDK7 (DC_50_ < 100 nM) Cyclin H (DC_50_ < 100 nM) MAT1 (DC_50_ < 100 nM) in Jurkat and OVCAR3 cells with 6‐h treatment	Jurkat, OVCAR3, SUDHL5, MOLT4, A549	Not reported, except for good tolerability	VHL	[114]
TZ1104	PROTAC	CDK7 (DC_50_ = 4.9 nM) in MV‐4‐11 cells with 4‐h treatment	MOLM13, THP‐1, HL‐60, CESS, RS4;11	Yes, tumor‐inhibitory activity against MV‐4‐11 xenograft model	VHL	[303]
CXJ2080	PROTAC	CDK7 (DC_50_ = 0.9 nM) in MV‐4‐11 and RS4;11 cells with 6‐h treatment	MV‐4‐11, RS4;11, patient‐derived acute myeloid leukemia cells	Yes, tumor‐inhibitory activity against RS4;11, MV‐4‐11, and MOLM13 xenograft models	VHL	[303]
JH‐XI‐10‐02	PROTAC	CDK8 (DC_50_ ≈ 100 nM) in Jurkat cells with 24‐h treatment	Jurkat	Not reported	CRBN	[304]
SNX7886	PROTAC	CDK8 (DC_50_ < 30 nM) CDK19 (DC_50_ < 30 nM) Cyclin C (DC_50_ < 30 nM) in HEK293T cells with 24‐h treatment	HEK293T	Not reported	CRBN	[116]
THAL‐SNS‐032	PROTAC	CDK9 (DC_50_ < 250 nM) in MOLT4 cells with 1‐h treatment	MOLT4, MOLT16, HSB‐2, KOPT‐K1, P12‐ICHIKAWA, SUP‐T11, DND‐41, etc.	Not reported	CRBN	[305]
Compound 45	PROTAC	CDK9 (DC_50_ < 100 nM) in MDA‐MB‐231 cells with 4‐h treatment	MDA‐MB‐468, MDA‐MB‐231, BT‐549	Yes, tumor‐inhibitory activity against MDA‐MB‐231 xenograft model	CRBN	[306]
Compound C3	PROTAC	CDK9 (DC_50_ ≈ 1 nM) in NCI‐H69 cells with 8‐h treatment	NCI‐H69, NCI‐H146, NCI‐H446, NCI‐H524, DMS114	Yes, tumor‐inhibitory activity against NCI‐H446 and NCI‐H69 xenograft models	CRBN	[307]
dCDK9‐202	PROTAC	CDK9 (DC_50_ = 3.5 nM) in TC‐71 cell lines with 8‐h treatment	T778, A375, MES‐SA, MDA‐MB‐231, A673, NCI‐H226, NALM6, SJSA1, RH5, REH	Yes, tumor‐inhibitory activity against TC‐71 xenograft model	CRBN	[308]
dCDK9‐010	PROTAC	CDK9 (DC_50_ = 0.24 µM in SJSA1, 0.5 µM in NCI‐H226, and 1.4 µM in TC‐32) with 8‐h treatment Cyclin T1/T2 (codepletion reported)	NCI‐H226, TC‐32, SJSA1, U2OS, CADO‐ES1, HCT116, HT1080, U87, and several other TP53 wild‐type cancer cells	Yes, tumor‐inhibitory activity against NCI‐H226, TC‐32, and SJSA1 xenograft models	MDM2	[309, 310]
BSJ‐4‐116	PROTAC	CDK12 (DC_50_ < 1 µM) in Jurkat cells with 6‐h treatment	Jurkat, MOLT4, Kelly	Not reported	CRBN	[311]
Compound 7f	PROTAC	CDK4 (DC_50_ = 10.5 nM) CDK6 (DC_50_ = 2.5 nM) in Jurkat cells	Jurkat	Not reported	CRBN	[318]
BSJ‐05‐017	PROTAC	CDK4/6 (DC_50_ < 10 nM) in MOLT4 cells with 5‐h treatment	MCF7, MOLT4	Yes, tumor‐inhibitory activity against MCF7 xenograft models	VHL	[313]
Compound 3	PROTAC	CDK2/4/6 (DC_50_ < 500 nM) in B16F10 and A375 cells with 12‐h treatment	B16F10, A375	Not reported	CRBN	[323]
ZLC491	PROTAC	CDK12 (DC_50_ = 32 nM) CDK13 (DC_50_ = 28 nM) in MDA‐MB‐231 cells with 15‐h treatment	MDA‐MB‐231, HCC38, MDA‐MB‐436	Yes, tumor‐inhibitory activity against MDA‐MB‐231 xenograft models	CRBN	[316]
YJ1206	PROTAC	CDK12/13 (DC_50_ ≈ 5 nM) in VCaP cell with 4‐h treatment	22Rv1, VCaP	Yes, tumor‐inhibitory activity in the WA74 xenograft model	CRBN	[315]
Compound F3	PROTAC	CDK9 (DC_50_ = 33 nM) CDK2 (DC_50_ = 62 nM) in PC‐3 cell with 4‐h treatment	PC‐3	Not reported	CRBN	[317]
HQ461	Molecular glue	Cyclin K (DC_50_ = 132 nM) in HEK293T cells with 24‐h treatment CDK12 (codepletion reported)	HEK293T, A549, HCT116	Not reported	CRL4^DDB1^	[327]
CR8	Molecular glue	Cyclin K (DC_50_ < 1 µM) in HEK293T cells with 1‐h treatment	HEK293T, MOLT4, etc.	Not reported	CRL4^DDB1^	[324]
dCeMM2 dCeMM3 dCeMM4	Molecular glue	Cyclin K (DC_50_ < 2.5, 7, 3.5 µM, respectively) CDK12/13 (codepletion reported) in KBM7 cells with 5‐h treatment	KBM7, HCT116, MV‐4‐11, etc.	Not reported	CRL4^DDB1^	[329]
NCT02	Molecular glue	Cyclin K (DC_50_ < 0.5 µM) CDK12/13 (codepletion reported) in TSC03 with 6‐h treatment	Patient‐derived colorectal cancer spheroid cultures	Not reported	CRL4^DDB1^	[328]
SR‐4835	Molecular glue	Cyclin K (DC_50_ ≈ 100 nM) in A375 cells with 2‐h treatment	A375, Colo829, WM164, WM983A, WM983B, TSC03, etc.	Yes, tumor‐inhibitory activity against TSC03 xenograft model	CRL4^DDB1^	[325, 328]
Compound 26	HEMTAC	CDK4 (DC_50_ = 26 nM) CDK6 (DC_50_ = 19 nM) in B16F10 cells with 24‐h treatment	B16F10, A375, HepG2, MDA‐MB‐231, A549	Yes, tumor‐inhibitory activity against B16F10 xenograft model	HSP90‐associated E3 complex	[333]
LL‐K9‐3	HyT	CDK9 (DC_50_ = 662 nM) Cyclin T1 (DC_50_ = 589 nM) in 22RV1 cells with 24‐h treatment	22RV1	Not reported	No	[335]
LL‐CDK9‐12	HyT	CDK9 (DC_50_ = 362 nM) Cyclin T1 (DC_50_ = 683 nM) in 22RV1 cells with 24‐h treatment	22RV1	Not reported	No	[336]
AZ‐9	HyT	CDK9 (DC_50_ = 407 nM) Cyclin T1 (DC_50_ = 1215 nM) in HCT116 cells (exposure time not specified)	HCT116, HT‐29, DLD‐1, RKO, MOLM13	Yes, tumor‐inhibitory activity against HCT116 xenograft model	No	[337]
Degrader 10	ATTEC	CDK9 (DC_50_ ≈ 100 nM) Cyclin T1 (DC_50_ ≈ 100 nM) in U‐2932 cells with 24‐h treatment	U‐2932, MV‐4‐11	Yes, tumor‐inhibitory activity against MV‐4‐11 xenograft model	No	[338]
CDK‐TCIP1	TCIP	CDK9 BCL6	SUDHL5, KARPAS422, BCL6‐expressing diffuse large B‐cell lymphoma cells	Not reported	No	[17]
CDK‐TCIP2	TCIP	CDK9 BCL6	SUDHL5	Not reported, except for good tolerability and ability to suppress germinal center B cells in immunized mice	No	[17]
CDK‐TCIP3	TCIP	CDK12/13 BCL6	SUDHL5	Not reported	No	[17]

All agents listed are in preclinical development.

#### 5.1.2 Selective PROTACs Targeting Transcription‐Associated CDKs

CDK7‐selective PROTAC JWZ‐5‐13, developed from conjugation of a VHL‐recruiting ligand with a high‐affinity CDK7 inhibitor YKL‐5‐167, induced rapid (≤0.5 h) and efficient degradation of CDK7, with minimal off‐target effects on other CDKs [114, 301, 302]. TZ1104 and CXJ2080, another VHL‐recruiting CDK7 PROTACs, promoted destabilization of the entire CAK complex and exhibited antileukemia effect both in vitro and in vivo [303]. CDK8‐selective PROTAC JH‐XI‐10‐02 is a CRBN‐dependent degrader by appending a PEG_4_‐pomalidomide moiety to the CDK8/19 inhibitor JH‐VIII‐49 [304]. This compound achieved near‐complete CDK8 ablation without affecting CDK19. Representative CDK9‐targeting PROTACs include THAL‐SNS‐032 (a thalidomide and SNS‐032 conjugate) [305], compound 45 (a pyrazolopyrimidine and lenalidomide conjugate) [306], compound C3 (an AT‐7519 and CRBN ligand conjugate) [307], and dCDK9‐202 (a TX‐16 and SNS‐032 conjugate) [308]. THAL‐SNS‐032 and dCDK9‐202 induced efficient degradation of cellular CDK9 within 2 h and produced longer‐lasting transcriptional shutdown than occupancy‐based inhibition. Compound C3 eliminated CDK9 within 8 h at a dose of 1.0 nM in small cell lung cancer cells and exhibited potent antiproliferative activities at sub‐nanomolar concentrations (IC_50_ = 0.53–3.77 nM). Both compound 45 and compound C3 demonstrated antitumor activities in xenograft animal models. Remarkably, Guan et al. recently developed a first‐in‐class, selective MDM2‐recruiting P‐TEFb PROTAC degrader, dCDK9‐010 (an RG7388 and SNS‐032 conjugate), which induces proteasome‐dependent degradation of CDK9 and all cyclin T isoforms while concurrently stabilizing p53 [309, 310]. dCDK9‐010 demonstrated a favorable safety profile and selective antitumor efficacy in MDM2‐proficient and TP53‐wild‐type cancer models [309, 310]. CDK12‐selective PROTAC BSJ‐4‐116 was developed by connecting a fragment derived from the dual CDK12/13 inhibitor THZ531 to thalidomide [311]. BSJ‐4‐116 achieved robust (IC_50_ = 6 nM) and selective degradation of CDK12 in Jurkat cells at a concentration as low as 50 nM within 6 h, while sparing CDK13 and other kinases. Functional studies revealed that key molecular responses to BSJ‐4‐116 treatment include premature intronic polyadenylation, which causes transcriptional downregulation of DDR and long genes. BSJ‐4‐116 also effectively overcame resistance associated with covalent CDK12 inhibitors and showed strong synergy with PARP inhibitors, highlighting its therapeutic potential in DDR‐dependent cancers.

#### 5.1.3 Dual‐Targeting PROTACs of CDKs

Given functional redundancy among CDKs and drug resistance following isoform‐selective inhibition, dual‐targeting PROTACs of CDKs may enhance cytotoxicity and therapeutic efficacy. Indeed, a number of dual‐targeting PROTACs against CDK4/6, CDK12/13, CDK8/19, and CDK2/9 have been reported with good potency and selectivity (Table 2 and Figure S2) [116, 312, 313, 314, 315, 316, 317, 318]. Representative CDK4/6‐targeting PROTACs include BSJ‐03‐204 (palbociclib‐based, CRBN‐recruiting), BSJ‐05‐017 (palbociclib‐based, VHL‐recruiting), and compound 7f (YY173‐based, CRBN‐recruiting) [312, 313, 319]. In the context of acquired resistance to CDK4/6 inhibitors, several mechanisms have been implicated, including overexpression or activation of CDK2/cyclin E, as well as induced binding between CDK6 and the CDK inhibitor p18^INK4C^ [313, 320, 321, 322]. Hence, CDK4/6 degraders and CDK2/4/6 degraders may be applicable for preventing or overcoming treatment resistance. Remarkably, BSJ‐05‐017 induced near‐complete degradation of CDK4 and CDK6, strongly inhibited pRB phosphorylation and levels of cyclin A2 and cyclin E2, and exerted substantial antitumor effects in breast cancer models resistant to CDK4/6 inhibitors. Although compound 3 (a ribociclib‐derivative‐based, CRBN‐recruiting tri‐targeting degrader of CDK2/4/6) has been reported [323], its activity in the context of acquired drug resistance remains to be examined. Orally bioavailable CDK12/13‐dual‐targeting PROTACs include ZLC491, and YJ1206, both of which engage CRBN to achieve efficient degradation of both CDK12 and CDK13 at low‐nanomolar concentrations and demonstrate in vivo degradative and antitumor effects [315, 316]. Additionally, SNX7886 and compound F3 are a CDK8/CDK19 degrader and a CDK2/CDK9 degrader, respectively. SNX7886, a hybrid of CDK8/19 inhibitor BI1347 and pomalidomide, degraded CDK8 (90%), CDK19 (80%), and cyclin C at 30 nM; however, its cytotoxicity profile remains unclear [116]. Compound F3 is an FN‐1501‐based, CRBN‐recruiting PROTAC that effectively induced degradation of both CDK9 (DC_50_ = 33 nM) and CDK2 (DC_50_ = 62 nM) and disrupted proliferation and cell cycle progression of prostate cancer cells [317]. Collectively, these studies highlight the rapid expansion and therapeutic promise of selective and dual‐targeting CDK degraders in oncology.

### 5.2 Molecular Glue

Molecular glues constitute a unique class of small‐molecule degraders that induce de novo protein‐protein interactions between E3 ubiquitin ligases and non‐native substrates (neosubstrates). Unlike PROTACs, molecular glues generally lack a separable linker or ligand structure. Instead, their degradation mechanism stems from conformational remodeling of target protein surfaces to enable E3 ligase engagement and subsequent ubiquitin conjugation. One of the best characterized molecular glue targets is cyclin K, which is usually degraded via recruitment of CDK12/cyclin K or CDK13/cyclin K complexes to the CRL4^DDB1^ E3 ligase. Representative cyclin K‐targeting molecular glues with disclosed structures include HQ461, CR8, dCeMM2/3/4, NCT02, and SR‐4835 (Table 2 and Figure S3A) [324, 325, 326, 327, 328, 329, 330]. While most of these molecular glues selectively degraded cyclin K and disrupted CDK12/13 activities, NCT02 showed the activity to deplete cyclin K, CDK12, and CDK13. Given that cyclin K is essential for CDK12/13 kinase activity and that dual CDK12/13 inhibition elicits maximal cytotoxicity [183], these cyclin K‐targeting molecular glues, regardless of whether they possess intrinsic CDK12/13 inhibitor/degrader activity, inevitably impair the global CDK12/13 function. Remarkably, CT7439, a clinical‐stage CDK12/13 inhibitor and cyclin K‐targeting molecular glue (Figure 4B), is currently in Phase 1/2 trials for advanced solid tumors (active trial: NCT06600789). Beyond CDK12/13/cyclin K, CDK2/cyclin E has emerged as another molecular glue target of significant industrial interest; various CDK2‐directed molecular glues (e.g., MRT‐9643, MRT‐51443, KT‐200) and cyclin E1‐targeting molecular glues (e.g., MRT‐55811) are under development with clinical translation potential [331, 332].

### 5.3 HEMTAC

HEMTACs represent a class of novel targeted protein degradation modality that harnesses the intrinsic chaperone activity of HSP90 and its associated E3 ligases to expand the scope of degradable proteins. HEMTACs are heterobifunctional compounds that link a ligand for a POI to a high‐affinity HSP90‐binding moiety via a flexible linker. This architecture facilitates the formation of a POI–HEMTAC–HSP90/E3 complex, leading to ubiquitination and subsequent proteasomal degradation of the target protein (Figure 4A). To date, the HEMTAC approach has been implemented to degrade CDK4/6 (Table 2 and Figure S3B) [333]. Li et al. developed compound 26 as a prototype CDK4/6‐targeting HEMTAC, and demonstrated its potent activity in depleting CDK4 (DC_50_ = 26 nM; maximum degradation = 88%) and CDK6 (DC_50_ = 19 nM; maximum degradation = 92%) following 24‐h treatment in murine melanoma cells. Notably, this CDK4/6 HEMTAC also exhibited sub‐micromolar antiproliferative effects across multiple cancer cell lines, and significantly suppressed tumor growth in both zebrafish and mouse xenograft models with favorable tolerability.

### 5.4 HyT Degrader

HyT‐based degraders are composed of a ligand that selectively binds the POI, a flexible linker, and a hydrophobic moiety that mimics partially unfolded protein conformation (Figure 5). This structural mimicry facilitates recognition by either molecular chaperones or components of the autophagy machinery, thereby initiating degradation of the POI through proteasome‐ or autolysosome‐mediated pathways, respectively. Qiu et al. reported an adamantyl‐based degrader of CDK4/6, LPM3770277, which showed a micromolar activity to degrade both CDK4 and CDK6 via proteasome, as well as a putative process involving lysosome‐mediated autophagy [334]. Despite modest potency to induce CDK4/6 degradation, LPM3770277 demonstrated superior antitumor efficacy and safety as compared with its parental compound abemaciclib. Li et al. described the SNS‐032‐derived HyT degrader LL‐K9‐3, which selectively induced concurrent proteasomal degradation of the CDK9/cyclin T1 complex (DC_50_: 0.66 µM for CDK9; 0.59 µM for cyclin T1), leading to marked suppression of androgen receptor‐ and MYC‐driven transcriptional programs in prostate cancer cells [335]. Subsequent optimization of LL‐K9‐3 by incorporating an adamantyl‐based HyT yielded LL‐CDK9‐12, which demonstrated enhanced potency (DC_50_: 0.36 µM for CDK9; 0.68 µM for cyclin T1) and antiproliferative efficacy [336]. Further expanding this strategy, Zhong et al. engineered AZ‐9, an ATG101 (autophagy‐related protein 101)‐recruiting HyT degrader, by conjugating CDK inhibitor AT‐7519 to an S‐indacene‐based hydrophobic moiety [337]. S‐indacene is able to recruit ATG101, which is a component of autophagy initiator Unc‐51‐like kinase complex. Mechanistically, AZ‐9 selectively degrades CDK9 and cyclin T1 through recruiting ATG101 and activating the autophagy‐lysosome degradation pathway (DC_50_: 0.41 µM for CDK9; 1.21 µM for cyclin T1). Despite employing the pan‐CDK inhibitor AT‐7519 as its warhead, the degradation selectivity profile of AZ‐9 favors CDK9 over other cell cycle CDKs (CDK2, CDK4, and CDK6). Hence, AZ‐9 represents the first ATG101‐recruiting CDK9/cyclin T1 HyT, working through a unique mechanism involving autophagy‐lysosome‐dependent proteolysis. Based on the above‐mentioned examples (Table 2 and Figure S3C), HyT‐based CDK degraders demonstrate two notable characters: capability to simultaneously degrade CDKs and its cyclin partners, and involving both proteasome system and lysosomal system for target degradation.

### 5.5 ATTEC

ATTECs represent a class of precision autophagy modulators by tethering the POI to autophagosome protein microtubule‐associated protein 1 light chain 3 (LC3) for subsequent degradation through autophagy‐lysosome pathway (Figure 5B). Early success in the development of CDK‐targeting ATTECs (e.g., degrader 10) was made by connecting SNS‐032 to an optimized LC3B‐recruiting Coumarin analog, which led to formation of a ternary complex with CDK9/cyclin T1 and LC3B (Table 2 and Figure S3D) [338]. Similar to POI–PROTAC–E3 ligase complex, the CDK9/cyclin T1–ATTEC–LC3B complex then facilitated autophagic degradation of CDK9 and cyclin T1.

## 6 Targeted Protein Redistribution Technologies for CDK Modulation

**FIGURE 6:**
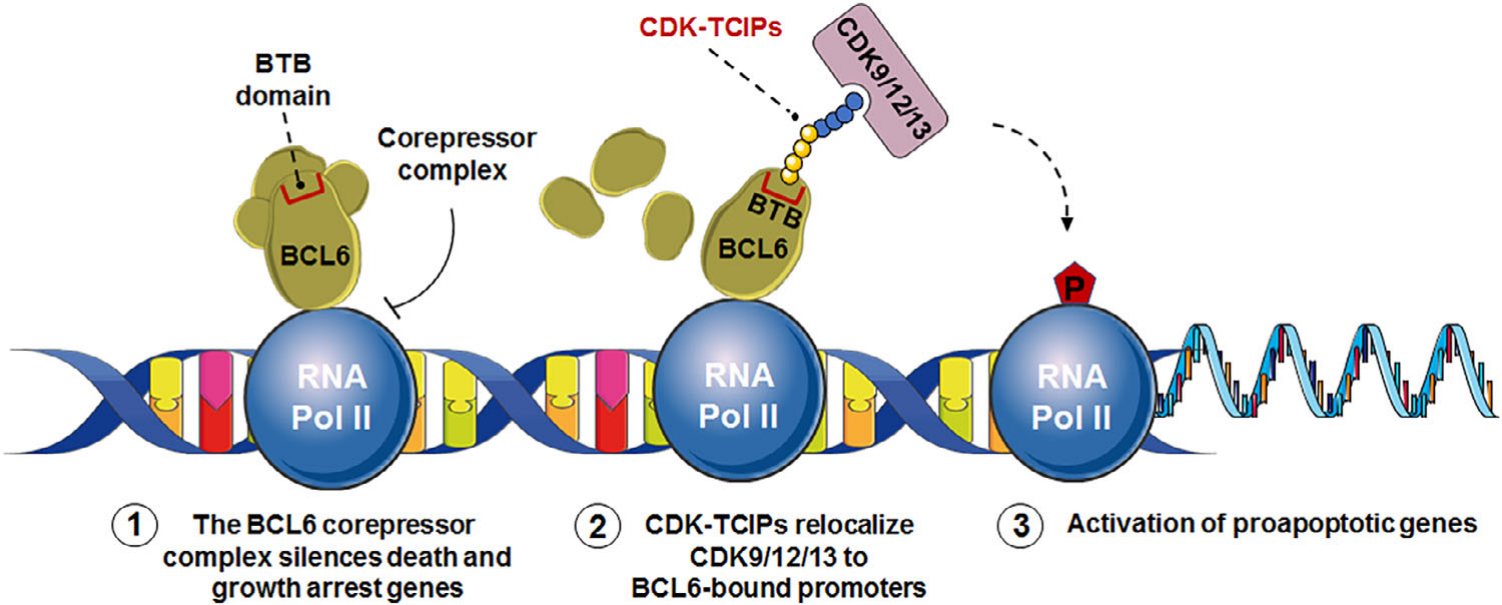
Working mechanisms of CDK‐TCIPs. CDK‐TCIPs target the BCL6 BTB domain to disrupt these repressive complexes, recruiting CDK9/12/13 to BCL6‐bound genomic loci. The mechanism triggers CDK‐mediated phosphorylation of Pol II, relieving transcriptional repression and activating proapoptotic target gene programs in BCL6‐addicted lymphoid malignance. Arrow denotes a regulatory event.

In contrast to targeting CDK through loss‐of‐function strategies, recent studies reported a novel gain‐of‐function strategy to turn CDK inhibitors into locus‐specific activators. Specifically, a class of bifunctional compounds, termed CDK‐transcriptional/epigenetic chemical inducers of proximity (CDK‐TCIPs), demonstrates a unique mode of action to redefine kinase targeting strategy [17]. The reported CDK‐TCIPs are bivalent molecules that tether an ATP‐competitive CDK inhibitor (e.g., SNS‐032 targeting CDK9) to a high‐affinity ligand (i.e., BI3812) targeting the BTB domain of a TF BCL6 (Table 2 and Figure S3E). CDK‐TCIPs spatially redirect kinase to specific genomic loci, and operate via a “catch‐and‐release” mechanism (Figure 6). For instance, CDK‐TCIP1 (BAK‐04‐021) has been shown to engage and guide CDK9 to the proximity of BCL6 target genes, while preserving its catalytic activity to phosphorylate Pol II. At the target loci (e.g., promoters repressed by BCL6), CDK9 can be released from the TCIP binding, unlocking its activity for Pol II phosphorylation and transcriptional elongation. As a result of CDK‐TCIP1 treatment, transcriptionally silenced BCL6 targets (e.g., proapoptotic genes such as PMAIP1/NOXA, BBC3/PUMA, and BIK) can be reactivated, selectively inducing apoptosis in BCL6‐dependent tumor cells, especially diffuse large B‐cell lymphoma. Further chemical optimization by incorporating a rigid diazaspirodecal linker led to the development of CDK‐TCIP2 (BAK‐04‐232) with significantly enhanced cellular potency and pharmacokinetic properties. Importantly, the CDK‐TCIP strategy demonstrates promising versatility, as evidenced by its successful adaptation to transcriptional CDKs such as CDK12/13. For instance, CDK‐TCIP3 (BAK‐04‐338) represents a selective CDK12/13‐targeting TCIP enabling redistribution of CDK12/13 to BCL6‐bound chromatin loci and reprogramming of transcription. Hence, advances in TCIP‐based approaches highlight the potential of chemically induced protein redistribution to rewire oncogenic transcriptional programs by hijacking CDKs as modular effectors for precision gene regulation.

## 7 Conclusion and Prospects

As reviewed in this article, CDKs and cyclins orchestrate cell cycle progression, gene expression, genome integrity, epigenetic regulation, metabolic reprogramming, immune response, and many other molecular processes underpinning cancer hallmarks. This functional pleiotropy establishes CDK/cyclin network as a nodal signaling hub in oncogenesis and highlights their promise as therapeutic targets. As a result, CDK/cyclin‐targeted therapy has been widely explored in human cancers. The successful clinical translation of CDK4/6 inhibitors has revolutionized the treatment landscape for hormone receptor‐positive, HER2‐negative metastatic breast cancer, establishing CDK/cyclin‐targeted therapy as a paradigm‐shifting approach in precision oncology. Importantly, recent advances in targeted protein degradation and targeted protein redistribution technologies further open up new avenues for the development of CDK/cyclin‐targeted therapy.

### 7.1 Emerging Paradigms and Future Directions

The expanding roles of CDKs and cyclins in human cancer highlight their intricate functions during cancer development and encourage the further evaluation of CDK/cyclin biology, particularly through the following emerging paradigms.

#### 7.1.1 Spatiotemporal Orchestration of CDKs Along With Cell Cycle Progression

Exemplified by CDK1 (G2/M checkpoint), CDK2 (G1/S and G2/M progression), and CDK4/6 (R point), prototypical cell cycle‐regulatory CDKs exert dynamic and cell cycle phase‐specific functions and regulatory impacts. Multilayered controls are involved: (a) stage‐specific cyclin availability and formation of distinct CDK/cyclin complexes, (b) posttranslational regulation of CDK catalytic activities including phosphorylation by CAK or SAPKs, and (c) subcellular localization including phase‐dependent organelle‐targeting, chromatin occupation and nuclear‐cytoplasmic shuttling. Of note, selective disruption of CDKs (e.g., CDK4/6) outside their established functional phases still exerts substantial effects on cellular processes and endomitosis [339]. Further elucidating the spatiotemporal regulation of CDKs will inform the better development of targeted therapeutic strategies.

#### 7.1.2 Coordinated Reprogramming of Cancer Metabolism by CDKs

In concert with their aberrant activities in cancer, CDKs rewire cellular metabolism to favor malignant progression mainly through kinase‐dependent and transcriptional mechanisms. Cell cycle‐regulatory CDKs (e.g., CDK1, CDK2, and CDK6) are able to directly phosphorylate metabolic enzymes to reshape bioenergetics [53, 72, 97], while transcription‐associated CDKs (e.g., CDK7, CDK8, CDK9, and CDK12) govern metabolic gene expression networks mainly via modulating Pol II activities [118, 131, 132, 188, 189, 340]. Cotargeting CDKs and metabolism vulnerabilities may create synthetic lethality against cancer, warranting systematic exploration and further investigation.

#### 7.1.3 Regulation of DDR and Genomic Integrity by CDKs and Cyclins

In addition to CDK1 and CDK2, which have been widely appreciated in DNA damage checkpoint control (not covered by this review), recent studies have revealed critical involvement of D‐type cyclins, CDK4/6, and many transcription‐associated CDKs in regulating DDR and genomic integrity. Interestingly, D‐type cyclins can be recruited to oxidized DNA lesions in a PCNA‐ and p21^Cip1^‐dependent, but CDK‐independent manner, where they limit mismatch repair during G0 and G1 phase [341]. Upon G1/S transition, timely degradation of D‐type cyclins then enables mismatch repair activity to efficiently correct DNA replication errors throughout S phase. Inactivation of CDK4/6 in cells under G2 arrest results in genomic duplication [342, 343]. Inactivation of CDK7/9/12/13 insults the proficient expression of DNA damage repair genes (e.g., BRCA1/2) [137, 181, 183, 184, 344, 345, 346, 347, 348, 349]. Hence, CDKs and cyclins serve as master regulators of DDR and damage repair pathways through a compound mechanism: D‐type cyclins restrict S‐phase‐specific functionality of mismatch repair; cell cycle‐regulatory CDKs govern replication stress surveillance and mitotic catastrophe prevention; and transcriptional CDKs maintain expression integrity of DDR genes. CDK inhibition may confer selective vulnerabilities (e.g., BRCAness) to PAPR inhibitors or other DNA‐damaging chemotherapeutic agents [350].

#### 7.1.4 Maintenance of Oncogenic Transcriptional Addiction by CDKs

Cancer cells usually rely on dysregulated transcriptional programs driven by oncogenic TFs and chromatin regulators (e.g., BRD4, p300, and histone deacetylases) to sustain their malignant phenotypes. A number of transcription‐associated CDKs, especially CDK7/8/9/12/13/19, have been reported to maintain the rewired oncogenic transcriptional networks through Pol II modulation and epigenetic mechanisms engaging enhancer/super‐enhancer regulation [351]. In parallel, cell cycle‐regulatory CDKs and atypical CDKs contribute to oncogenic transcription through multiple ways: (a) activation of downstream oncogenic TFs (e.g., E2F1, MYC, and FOXM1), (b) regulation of chromatin modifiers (e.g., DNA methyltransferases, and histone methyltransferases), and (c) noncanonical chromatin‐binding activities (e.g., CDK6). Hence, functional boundaries between transcription‐associated CDKs and cell cycle‐regulatory CDKs in sustaining malignant gene expression programs become blurred. Simultaneously targeting CDKs in conjunction with other anticancer agents likely mitigates the development of therapy resistance in part through suppressing the transcriptional adaption. Consequently, CDK‐targeted agents serve as transcriptional adjuvants to amplify the response and durability of conventional therapies.

#### 7.1.5 Regulation of Cancer Immunity by CDKs and Cyclins

CDKs and cyclins are increasingly recognized as important regulators of immunomodulation, modulating interactions between cancer cells and the immune microenvironment through their functional impacts on both cancer cells and noncancer compartments. In cancer cells, proficient expression of several CDKs (e.g., CDK2, CDK4/6, and CDK9) has been shown to repress endogenous retroviral expression, tumor antigen presentation, and IFN response, meanwhile promote the expression of diverse immune modulators especially PD‐L1. Inhibition of CDKs alters secretion of immunomodulatory cytokines from cancer cells to regulate the recruitment and functionality of immune cells (especially T cells and myeloid cells). Cancer cells with defective DDR, induced by CDK blockage, may provide new opportunity for adjuvant immune therapy [352]. In noncancer cells, targeted inhibition of CDKs also exerts immunomodulatory effects. For instance, CDK2/cyclin J promotes innate immune response through metabolic programming of macrophages; CDK4/6 inhibitors suppress the proliferation of regulatory T cells. Besides, CDK‐targeted agents may influence cancer immunity via senescence induction, metabolic/immunometabolic changes, and intratumoral microbes. Till now, the impact of CDK‐targeted agents on nonmalignant cellular compartments is poorly defined. The immune‐regulatory roles of CDKs in cancer represent a fertile ground for scientific investigations.

#### 7.1.6 Deciphering the Redundant and Unique Functions Among Homologous CDKs and Cyclins

A major challenge in CDK/cyclin biology is to distinguish functionally redundant from isoform‐specific roles among homologous CDKs and cyclins. This distinction is essential, as functional compensation and high sequence similarity often obscure unique functions. For instance, genetic ablation of CDK2 can be rescued by CDK1, and resistance to CDK4/6 inhibitors frequently converges on CDK2 activation driven by CCNE1/2 amplification or cyclin E upregulation. Compensatory activities have also been appreciated for other pairs, including CDK4 and CDK6, CDK11A, and CDK11B, as well as CDK12 and CDK13. To circumvent functional compensation, the development of dual‐targeting protein degraders or inhibitors will be valuable. Conversely, isoform‐specific functions have also been identified in several cases, such as CDK4 versus CDK6, CDK12 versus CDK13, the long and short isoforms of CDK9, cyclin T1 versus T2, and certain highly homologous cyclins. The overlapping temporal and spatial expression patterns of distinct CDK/cyclin pairs further complicate functional dissection. To address these challenges, developing isoform‐selective degraders or inhibitors is a promising strategy. Such tools would not only facilitate functional evaluation but also enable therapeutic interventions that target only disease‐relevant activities while sparing housekeeping functions of other homologs. A compelling example is the differential lineage dependency on CDK4 versus CDK6. Despite their functional redundancy, HER2‐positive breast cancer and prostate cells rely more on CDK4 than CDK6 [353], while hematopoietic cells are selectively sensitive to CDK6 depletion [354]. Hence, developing CDK4‐selective inhibitors may avoid the dose‐limiting hematologic toxicities caused by “dual” CDK4/6 inhibitors [355]. Moreover, meta‐analysis of real‐world clinical data indicates that the adverse effects of are CDK4/6 inhibitors significant yet different among agents: palbociclib and ribociclib exhibit higher hematological toxicity (manifesting as neutropenia and leukopenia), while abemaciclib preferentially causes gastrointestinal toxicity (diarrhea) [356, 357]. Although the mechanistic basis of lineage‐associated CDK4 or CDK6 dependency warrants further investigation, this concept has already begun to inform next‐generation drug design. Notably, atirmociclib (PF‐07220060) represents a next‐generation CDK4‐selective inhibitor, showing improved safety and anticancer efficacy [358]. Together, further dissecting the redundant and unique functions of homologous CDKs and cyclins will provide critical insight into both fundamental biology and precision therapeutics.

#### 7.1.7 Expanding Research Into Understudied CDKs and Cyclins

Notably, a subset of CDKs, particularly atypical CDKs, constitutes part of the dark kinome with significant knowledge gaps. Since orphan kinases such as CDK15 and CDK17 lack conventional cyclin partners, they may possess noncatalytic scaffolding functions. Furthermore, the functional significance of CDK3, CDK10, and CDK11 in oncogenesis remains inadequately understood. Elucidating the biological mechanisms of these understudied CDKs could provide valuable insights into cancer pathogenesis and inform the development of targeted therapeutic strategies. Besides, the noncanonical activities of CDKs and cyclins (i.e., CDK‐independent function of cyclins and vice versa) await further investigation.

#### 7.1.8 Advantages and Challenges in Targeted Protein Degradation

Targeted protein degradation employs proteolysis‐based technologies such as PROTACs, HEMTACs, HyTs, molecular glues, and ATTECs. Their corresponding typical degraders were summarized in Table 2. Beyond the approaches reviewed here, DNA framework‐based PROTACs targeting CDK9 and CDK6 have been developed with favorable selectivity [359]. Peptide‐directed degradation of CDK5 through chaperone‐mediated autophagy has also been reported in a study related to stroke intervention [360]. These approaches may offer advantages over conventional inhibitors by overcoming kinase domain mutation‐driven resistance and eliminating kinase‐independent scaffolding functions of CDKs. Notably, CDK inhibitors and their related degraders exhibit distinct target profiles. Through structural optimization, several isoform‐selective degraders have been reported. For example, PROTACs targeting CDK4, CDK6, CDK7, CDK9, CDK12, and CDK13 have emerged as valuable tools to dissect isoform‐specific roles in cancer. Despite the encouraging efficacy and immense potential of the CDK/cyclin‐targeting degraders reviewed herein, their clinical translation faces substantial challenges, many of which are common to targeted protein degradation modalities. These include poor bioavailability, off‑target effects, an incomplete understanding of E3 ligase biology, limited cellular selectivity, and design/manufacturing complexities [18, 361, 362, 363, 364, 365, 366]. Specially, increased size of heterobifunctional molecules like PROTACs, HEMTACs, and ATTECs hinders passive diffusion across plasma membranes. Increased hydrophobicity of PROTACs and HyTs yields poor aqueous solubility. Meanwhile, their bifunctional design also complicates the absorption, distribution, metabolism, and excretion profiles [367]. Hence, advances in physicochemical property optimization, linker design, innovative formulations, prodrug strategies, and nanoparticle delivery represent the most promising approaches to overcome these bioavailability limitations [367, 368, 369, 370]. Many heterobifunctional molecules rely on E3 ligase for target protein degradation, yet only a handful of the E3 ligases in the human genome have been successfully engaged to date, with CRBN and VHL being the predominantly hijacked ones. Expanding this E3 ligase repertoire by identifying and characterizing novel E3 ligases will therefore be highly valuable [365]. As these degraders advance toward clinical use, it will become increasingly important to characterize the off‐target effects and to ensure specificity and safety. Indeed, many CRBN‐recruiting ligands suffer from unintended neosubstrate degradation (e.g., GSPT1, ZFP91, IKZF1/3), along with nondegraded interactomes [371], which may cause severe toxicity and adverse effects. Recently, Shrestha et al. developed ProtacID, a proximity‐dependent biotinylation‐based approach that identifies PROTAC‐proximal proteins in living cells, enabling the detection of nondegraded protein binding for VHL‐ and CRBN‐recruiting PROTACs [372]. To address cellular selectivity and efficacy challenges, PROTACs may have the adaptability by substituting ubiquitous E3 ligase (e.g., CRBN, VHL) binders with alternative ligands for cancer‐specific E3 ligases (e.g., MDM2), or by swapping an inefficient binder to a recruiter of a new E3 ligase (e.g., DCAF1) [309, 310, 373]. In the context of targeted therapy, E3 ligase expression heterogeneity across tumors will determine the optimal ligase choice for a given indication. Additionally, prodrug approaches, drug–biomacromolecule conjugates, and nanoparticle‐based delivery systems may provide a general solution for enhancing targeting precision and therapeutic efficiency [369, 370, 374, 375]. Encouraged by the development of several blood–brain barrier‐penetrant PROTACs and HyTs [376, 377, 378], more efforts are needed to deliver CDK/cyclin‐targeting protein degraders across the blood–brain barrier for the treatment of central nervous system tumors (e.g., glioblastoma) [379], as well as the blood‐testis barrier for testicular tumors. In contrast to heterobifunctional molecules, molecular glues are considered advantageous in more drug‐like properties, such as smaller molecular size, improved bioavailability, and simplified pharmacokinetics. Nevertheless, molecular glues face critical barriers: no universal design principles exit, and their application remains restricted to cyclin K, CDK12/13, or CDK2 degradation via select E3 ligases. Currently, the specificity and selectivity of glue‐like molecules are difficult to predict, given the sporadic and often serendipitous nature of their discovery. A high‐throughput ligand diversification coupled with a readout of targeted protein degradation offers a feasible route to prospectively identify structural modifications that convert conventional ligands into glue‐like molecules [380]. Further interdisciplinary innovation integrating computational prediction tools and artificial intelligence‐driven drug design may help unlock novel protein degraders [381, 382].

#### 7.1.9 Resistance Mechanisms in Targeted Protein Degradation and Potential Counterstrategies

Given the high dependency of targeted protein degradation modalities on E3 ligase and their functional complexes, reduced expression or genomic inactivation of E3 ligase itself, or of core components of the E3 ligase complex, renders cells less responsive to the relevant degraders [309, 310, 329, 366, 373, 383, 384]. Using a functional genomics approach, Shirasaki et al. reported that resistance to CRBN‐ or VHL‐based degraders converged on disruption of E3 ligase complex function rather than cellular adaptation to target degradation [385]. Key genes whose loss of function contributes to CRBN‐based PROTAC resistance include CRBN itself, DDB1, UBE2G1, and COP9 signalosome complex genes (e.g., COPS7B, COPS2, COPS3, COPS8); inactivated genes accounting for VHL‐based PROTAC resistance include VHL itself, CUL2, RBX1, ELOB, ELOC, UBE2R2, and COP9 signalosome genes (COPS7B, COPS8). Similarly, in the case of molecular glues, hotspot mutations associated with resistance are enriched in the substrate receptors of hijacked ligases, converging on altered ternary complex assembly [386]. Hence, E3 ligase expression and the functionality of the E3 ligase complex serve as straightforward biomarkers for degrader efficacy. Beyond E3 ligase‐associated factors, recent studies have revealed that the drug efflux pumps ABCB1 and ABCC1 mediate both intrinsic and acquired resistance to various PROTACs [387, 388, 389], suggesting that active efflux limits PROTAC efficacy in cancer cells. Additional resistance‐related factors involve genes in apoptosis and mTOR signaling pathways [387]. To counter these resistance mechanisms, potential strategies include combination therapy with BCL2 homology 3 mimetics (e.g., BCL2 or MCL1 inhibitors), mTOR inhibitors, or efflux pump inhibitors. Moreover, due to the mechanistic distinctions among different protein degradation strategies, acquired resistance to a specific modality (e.g., E3 ligase mutation or inactivation) may potentially be overcome by switching to a mechanistically different agent (such as a lysosome‐dependent degrader), as long as cancer cells retain dependence on target CDKs for survival and proliferation. Sequential administration of degraders made employing different E3 recruiters or mechanistically distinct degradation pathways likely minimizes the risk of acquired resistance [366, 373].

#### 7.1.10 Emerging Strategies and Challenges in Targeted Protein Redistribution

Targeted protein redistribution mainly uses the TCIP technique that leverages bifunctional molecules to relocate transcription‐associated CDKs (e.g., CDK9/12/13) to noncanonical binding sites. Current TCIPs are primarily reported to induce proximity between CDK9/12/13 and BCL6, but future efforts could expand to redirecting other CDKs to broader genomic loci. Of note, a similar strategy (inducing proximity between CDK9 and EZH2) has shown early promise in triggering DNA damage and antitumor effects [390]. Due to their bifunctional nature, TCIPs likely face intrinsic translational challenges similar to those of protein degraders. However, their resistance mechanisms remain unexplored. Future studies may examine whether they involve drug efflux pumps or apoptosis evasion. Other key hurdles in TCIP development include preserving CDK catalytic activity and broadening the scope of druggable targets. Alternatively, targeted protein redistribution may be achieved by modulating domain interactions to reconfigure the assembly of CDK‐epigenetic machinery complexes, or by sequestering CDKs in repressive chromatin domains, cytoskeletal structures, or organelles.

### 7.2 Key Takeaways

Collectively, the pervasive dysregulation of CDK/cyclin networks in cancer, driven by genomic alterations or epigenetic mechanisms, creates a permissive environment for malignant transformation and progression. While CDKs, cyclins, and their functional complexes are cornerstones of cell cycle control and transcriptional regulation, their diverse roles including noncanonical functions represent vast understudied territories and emerging frontiers in cancer biology. Future studies may prioritize elucidating CDK/cyclin‐immune interactions, CDK/cyclin‐metabolism crosstalk, CDK/cyclin‐driven oncogenic transcriptional addiction, and CDK/cyclin‐associated synthetic lethal strategies. Deciphering the redundant versus unique functions of homologous CDKs and cyclins will guide the development of isoform‐selective or dual‐targeting approaches. Targeted protein degradation and targeted protein redistribution represent promising directions for next‐generation therapies. Priority areas include establishing robust design principles, developing novel E3 ligase platforms, improving the target specificity and cell selectivity, and optimizing bioavailability and delivery efficiency.

## Author Contributions

**Suya Zheng**: methodology, visualization, writing – original draft, and writing – review and editing. **Zhipeng Shen**: methodology, writing – review and editing, and funding acquisition. **Yuanfang Wu**: methodology, visualization, validation, and writing – review and editing. **Xingze Huang**: methodology, visualization, and validation. **Zhongyuan Zhang**: methodology, validation, and writing – review and editing. **Jiangyu Liu**: visualization. **Qichun Wei**: validation, writing – review and editing, and funding acquisition. **Wenhao Chen**: writing – review and editing and funding acquisition. **Ye Chen**: conceptualization, methodology, visualization, writing – original draft, writing – review and editing, supervision, and funding acquisition. **Liang Xu**: conceptualization, methodology, writing – original draft, writing – review and editing, supervision, and funding acquisition. All authors have read and approved the final manuscript.

## Funding

This work is funded by the National Natural Science Foundation of China (32270746 to L.X., 82203415 to Y.C., 82372668 to W.C.), the Natural Science Foundation of Zhejiang Province (LZ24H160004 to Y.C., LMS26H160001 to Z.S.), the National Key Research and Development Program of China (2022YFE0107800 to Q.W.), Fundamental Research Funds for the Central Universities (226‐2025‐00101 to L.X.), ZJU‐GENSCI Children's Health Research & Development Center (ZJU‐GENSCI2024YY003 to Z.S.), and Medical Interdisciplinary Innovation Program 2024, Zhejiang University School of Medicine (to W.C. and L.X.).

## Ethics Statement

The authors have nothing to report.

## Conflicts of Interest

The authors declare no conflicts of interest.

## Supporting information




**Supporting File 1**: mco270898‐Sup‐0001‐SuppMat.docx

## Data Availability

Genomic and copy number data related to the cancer samples analyzed in this study were retrieved from cBioPortal (https://www.cbioportal.org/) by 2026‐May. Information related to clinical trials was obtained from ClinicalTrials.gov by 2026‐May. Kinase domain annotations were sourced from the Conserved Domain Database (https://www.ncbi.nlm.nih.gov/cdd).
