# Supplementary material for: Cyclins and Cyclin‐Dependent Kinases: Structure, Biological Functions, and Innovative Targeting Strategies in Cancer
Source: MedComm (2020). 2026 Aug 16;7(9):e70898. doi: 10.1002/mco2.70898 (PMC13478576; doi:10.1002/mco2.70898)
Supplement: Supplementary file 1 [file MCO2-7-e70898-s001.docx]

**Cyclins and cyclin-dependent kinases: structure, biological functions and innovative targeting strategies in cancer**

Suya Zheng^1,2,3,#^, Zhipeng Shen^2,3,#^, Yuanfang Wu^2,3,#^, Xingze Huang^4^, Zhongyuan Zhang^2,3^, Jiangyu Liu^2,3^, Qichun Wei^1,5^, Wenhao Chen^3,6,^*, Ye Chen^2,3,7,^*, Liang Xu^1,4,7,^*

^#^Equal contribution; *Correspondence.

**Supporting Information**

**Supplementary Figure S1.** Chemical structures of CDK inhibitors that have received regulatory approval.

**Supplementary Figure S2.** Chemical structures of representative CDK/cyclin-targeting PROTACs.

**Supplementary Figure S3.** Chemical structures of representative CDK/cyclin-targeting degraders and relocalization agents.

**Supplementary Table S1.** Copy number variations of CDKs and cyclins across human cancers (TCGA pan-cancer cohort via cBioPortal).

**Supplementary Table S2.** List of hotspot mutations and recurrent mutational events affecting CDK and cyclin genes.

**Supplementary Figures and legends**

**Supplementary Figure S1. Chemical structures of CDK inhibitors that have received regulatory approval.**

**Supplementary Figure S2. Chemical structures of representative CDK/cyclin-targeting PROTACs.** E3 ligase-recruiting ligands are shown in light blue, while the corresponding target protein binders are shown in deep pink.

**Supplementary Figure S3. Chemical structures of representative CDK/cyclin-targeting degraders and relocalization agents.** (A) Representative molecular glues targeting CDK/cyclin proteins. (B) A HEMTEC compound targeting CDK4/6. (C) Representative HyT molecules targeting CDK/cyclin proteins. (D) An ATTEC compound targeting CDK9/cyclin T1. (E) Representative CDK-TCIPs.

**Supplementary Table S1. Copy number variations of CDKs and cyclins across human cancers (TCGA pan-cancer cohort via cBioPortal).**

| Gene | Occurrence | | Overall incidence rate  (of 10967 cases) |
| --- | --- | --- | --- |
|  | **Amplification** | **Deletion** |  |
| CDK1 | 69 | 20 | 0.0081 |
| CDK2 | 88 | 4 | 0.0084 |
| CDK3 | 173 | 8 | 0.0165 |
| CDK4 | 251 | 1 | 0.0230 |
| CDK5 | 113 | 38 | 0.0138 |
| CDK6 | 206 | 13 | 0.0200 |
| CDK7 | 10 | 77 | 0.0079 |
| CDK8 | 76 | 37 | 0.0103 |
| CDK9 | 59 | 11 | 0.0064 |
| CDK10 | 39 | 128 | 0.0152 |
| CDK11A/B | 60 | 87 | 0.0134 |
| CDK12 | 278 | 19 | 0.0271 |
| CDK13 | 72 | 16 | 0.0080 |
| CDK14 | 176 | 17 | 0.0176 |
| CDK15 | 60 | 27 | 0.0079 |
| CDK16 | 91 | 53 | 0.0131 |
| CDK17 | 53 | 6 | 0.0054 |
| CDK18 | 240 | 7 | 0.0225 |
| CDK19 | 48 | 103 | 0.0138 |
| CDK20 | 32 | 15 | 0.0043 |
| CABLES1 | 175 | 15 | 0.0173 |
| CABLES2 | 250 | 3 | 0.0231 |
| CCNA1 | 56 | 79 | 0.0123 |
| CCNA2 | 18 | 28 | 0.0042 |
| CCNB1 | 9 | 79 | 0.0080 |
| CCNB2 | 20 | 33 | 0.0048 |
| CCNB3 | 81 | 57 | 0.0126 |
| CCNC | 36 | 98 | 0.0122 |
| CCND1 | 620 | 9 | 0.0574 |
| CCND2 | 197 | 15 | 0.0193 |
| CCND3 | 198 | 8 | 0.0188 |
| CCNE1 | 410 | 14 | 0.0387 |
| CCNE2 | 376 | 5 | 0.0347 |
| CCNF | 80 | 18 | 0.0089 |
| CCNG1 | 69 | 37 | 0.0097 |
| CCNG2 | 51 | 16 | 0.0061 |
| CCNH | 6 | 62 | 0.0062 |
| CCNI | 56 | 16 | 0.0066 |
| CCNI2 | 54 | 33 | 0.0079 |
| CCNJ | 18 | 30 | 0.0044 |
| CCNJL | 68 | 23 | 0.0083 |
| CCNK | 46 | 44 | 0.0082 |
| CCNL1 | 314 | 9 | 0.0295 |
| CCNL2 | 60 | 87 | 0.0134 |
| CCNO | 36 | 67 | 0.0094 |
| CCNP | 177 | 8 | 0.0169 |
| CCNQ | 178 | 29 | 0.0189 |
| CCNT1 | 35 | 12 | 0.0043 |
| CCNT2 | 21 | 31 | 0.0047 |
| CCNY | 80 | 10 | 0.0082 |
| CCNYL1 | 54 | 30 | 0.0077 |
| CCNYL2 | 66 | 25 | 0.0083 |
| CNPPD1 | 30 | 40 | 0.0064 |
| CNTD1 | 44 | 22 | 0.0189 |

**Supplementary Table S2. List of hotspot mutations and recurrent mutational events affecting CDK and cyclin genes.**

| **Gene** | **Target site** | **Mutation** | **Disease** | **Occurrence** |
| --- | --- | --- | --- | --- |
| *CDK4* | K22 | K22M | Skin cutaneous melanoma | 4 |
|  |  | K22Q | Skin cutaneous melanoma | 1 |
|  | R24 | R24C | Skin cutaneous melanoma | 1 |
|  |  | R24H | Skin cutaneous melanoma | 1 |
|  |  | R24L | Lung adenocarcinoma, Skin cutaneous melanoma | 3 |
|  |  | R24S | Skin cutaneous melanoma | 1 |
| *CDK11B* | N37 | N37Ifs*17 | Stomach adenocarcinoma, Colorectal adenocarcinoma, Endometrial carcinoma | 4 |
|  |  | N37K | Endometrial carcinoma | 1 |
|  |  | X37_splice | Colorectal adenocarcinoma | 1 |
| *CDK19* | R254 | R254Q | Colorectal adenocarcinoma | 1 |
|  |  | R254W | Colorectal adenocarcinoma, Endometrial carcinoma | 4 |
| *CCND1* | T286 | T286A | Uterine corpus endometrial carcinoma | 1 |
|  |  | T286I | Uterine corpus endometrial carcinoma | 6 |
|  |  | T286R | Uterine corpus endometrial carcinoma | 1 |
|  | P287 | P287H | Uterine corpus endometrial carcinoma | 2 |
|  |  | P287L | Uterine carcinosarcoma, Head and neck squamous cell carcinoma | 2 |
|  |  | P287R | Bladder urothelial carcinoma | 1 |
|  |  | P287S | Endometrial carcinoma | 2 |
|  |  | P287T | Endometrial carcinoma, Cervical squamous cell carcinoma | 3 |
| *CCNQ* | K141 | K141Sfs*14 | Lung adenocarcinoma | 1 |
|  |  | X141_splice | Lung adenocarcinoma, Skin cutaneous melanoma | 4 |
| *CCNT1* | X727 | *727Kext*12 | Acute myeloid leukemia, Breast invasive carcinoma, Ovarian serous cystadenocarcinoma | 5 |
| *CCNT2* | R255 | R255* | Colorectal adenocarcinoma, Endometrial carcinoma | 4 |
|  |  | R255L | Glioblastoma multiforme | 1 |
| *CCNYL1* | R196 | R196C | Endometrial carcinoma | 5 |

Footnote: The “Occurrence” column values represent patient numbers within the TCGA pan-cancer cohort.
